# Supplementary material for: NoLiTiA: An Open-Source Toolbox for Non-linear Time Series Analysis
Source: Front Neuroinform. 2022 Jun 24;16:876012. doi: 10.3389/fninf.2022.876012 (PMC9263366; doi:10.3389/fninf.2022.876012)
Supplement: Supplementary file 1 [file Data_Sheet_1.PDF]

NOLITIA

A Nonlinear Time Series Analysis Toolbox

User Manual

Version 1.2

Immo Weber

Immo.weber@systemsneuroscience.de

CLINICAL SYSTEMS NEUROSCIENCE

Table of Contents

1. Introduction 5

2. Installation 6

3. General Workflow 6

3.1. Overview 6

3.1.1. Load Data 6

3.1.2. Prepare Data (optional) 7

3.1.3. Choose Method(s) 7

3.1.4. Define/Optimize Embedding Parameters (optional) 7

3.1.5. Define Method-Specific Parameters (optional) 7

3.1.6. Calculate 7

3.1.7. Plot Results 7

3.1.8. Save Results 8

3.2. GUI 8

3.2.1. Overview 8

3.2.2. Workflow 9

3.3. Batch-Editor 12

3.3.1. Overview 12

3.3.2. Workflow 13

3.4. Plotting Tool 14

3.4.1. Workflow 14

3.4.2. Topographic Representation of EEG or MEG-Data 15

3.5. Statistics GUI 18

3.6. Custom-made Scripts 20
3.6.1. Overview 20
3.6.2. Workflow 21
4. Table of Functions 22
4.1. Nonlinear Dynamics Measures 22
4.1.1. Correlation Dimension 22
4.1.2. Lyapunov Exponent 24
4.1.3. Ragwitz Estimator 26
4.1.4. False Nearest Neighbours 27
4.1.5. Space-Time Separation Plot 29
4.1.6. Time Inversion 30
4.1.7. Hurst Exponent 31
4.1.8. Detrended Fluctuation Analysis 32
4.1.9. Unstable Periodic Orbit Transform 34
4.2. Recurrence Measures 36
4.2.1. Recurrence Plot 36
4.2.2. Joint Recurrence Plot 39
4.2.3. Cross Recurrence Plot 41
4.2.4. Recurrence Frequencies_over range of neighbourhoods 43
4.2.5. Windowed Recurrence Frequencies 45
4.3. Information Theoretic Measures 47
4.3.1. Shannon Entropy (Bin Estimator) 47

4.3.2. Differential Entropy (Kozachenko's Estimator) ... 48

4.3.3. Mutual Information (Bin Estimator) ... 49

4.3.4. Mutual Information (Kraskov's Estimator) ... 50

4.3.5. Mutual Information Matrix (Bin & Kraskov) ... 52

4.3.6. Auto-Mutual Information (Bin Estimator) ... 53

4.3.7. Auto-Mutual Information (Kraskov's Estimator) ... 54

4.3.8. Active Information Storage ... 55

4.4. Related ... 56

4.4.1. Phase Space reconstruction ... 56

4.4.2. Autocorrelation ... 58

4.4.3. Neighbours Distance (mex) ... 58

4.4.4. Optimize Embedding Parameters ... 59

4.4.5. Optimize Number of Bins for Histogram ... 61

4.4.6. Prepare Data ... 61

4.5. Data ... 62

4.5.1. Lorenz Data Generation ... 62

4.5.2. Sinusoid Generation ... 63

4.5.3. Logistic Map ... 64

4.5.4. Lorenz Data ... 64

4.6. Statistics ... 64

4.6.1. Generation of Surrogates ... 64

4.6.2. Monte-Carlo Test ... 65

4.6.3. Cluster-based permutation analysis 67

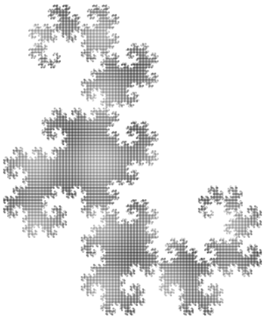

Marburg, 2018

1. Introduction

NoLiTiA is a free open-source Matlab®-Toolbox for the anaylsis of time series with possible nonlinear origin i.e. the observed data was generated by a nonlinear system. A system is nonlinear if a change of its input is non-proportional to its output. The toolbox is designed with the intention to be both flexible for the experienced user and intuitive for beginners. The range of available methods originate from three main fields: 1) (classic) nonlinear dynamics, 2) recurrence quantification, 3) information theory (Fig. 1).

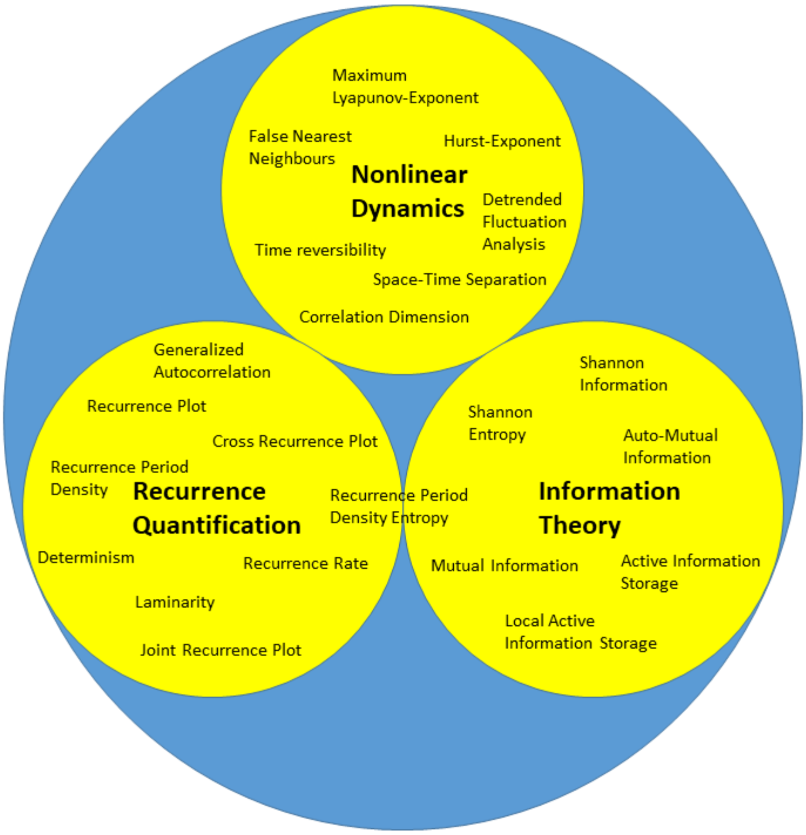

Figure 1: Topics covered by NoLiTiA.

2. Installation

To install the toolbox, download it from www.nolitia.com and simply copy the complete folder of NoLiTiA onto your computer. Finally, run the “install_nolitia.m” script within Matlab. NoLiTiA was developed using Matlab 2016b. Compatibility with older Matlab versions cannot be guaranteed.

3. General Workflow

3.1. Overview

The toolbox offers three distinct ways to analyse data, depending on the degree of needed flexibility, experience in programming and ease of use: 1) graphical user interface (GUI), 2) batch-editor, 3) custom-made Matlab-scripts.

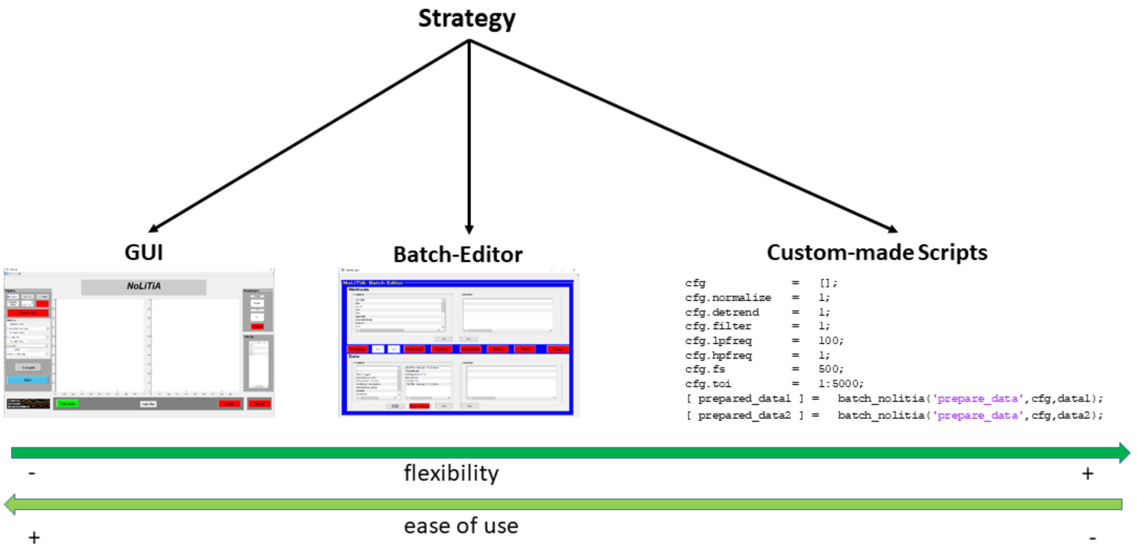

Figure 2: Analysis pathways.

No matter which of the three options the user chooses, all of them share more or less the same workflow:

3.1.1. Load Data

Depending on the user’s analysis pathway, data may either be loaded from a file or from workspace.

3.1.2. Prepare Data (optional)

NoLiTiA offers the possibility to pre-process your data. Options include the definitions of a time-region of interest, detrending, normalization and filtering (for filtering the signal processing toolbox needs to be installed). See Table 53 in section 3 for details.

3.1.3. Choose Method(s)

Choose methods from the three topics: 1) (classic) nonlinear dynamics, 2) recurrence based methods, 3) information theoretic measures. See section 4 for details.

3.1.4. Define/Optimize Embedding Parameters (optional)

For many methods, it is necessary to define embedding parameters (dim=dimension, tau=embedding delay) for state-space reconstruction. The three pathways offer either to define them ad-hoc by the user or to optimize them based on two different approaches ('deterministic', 'markov'). The optimization procedure should be chosen depending on whether your data was generated by a deterministic or a stochastic process (see section 3 for details).

3.1.5. Define Method-Specific Parameters (optional)

Every method has at least one parameter, which may be specified by the user. If a parameter is left unspecified, the default value is applied (see section 4 for a list of all parameters including default values).

3.1.6. Calculate

Run the analysis.

3.1.7. Plot Results

Depending on your analysis path, results are either plotted automatically (GUI), or the user may optionally choose to do so (batch, custom-made scripts, see section 3.3 and 3.6).

3.1.8. Save Results

You should be aware that results are not automatically saved. Either push the save button in the GUI or Batch-editor, or use the Matlab-command ‘save’ to save results.

3.2. GUI

3.2.1. Overview

The graphical user interface (GUI) is intended to be the most beginner’s friendly option for analysis. The GUI can be invoked by typing “nolitia_gui” in the command window (Fig. 3). The interface is composed of four main regions: on the left side, the user loads the input data, chooses whether and how to pre-process, specifies analysis methods, generates surrogate data and enters batch-mode. On the right side, the user may enter embedding parameters (dimension and tau) *ad-hoc* or choose to optimize them using two different approaches (see section 4.4.4). Method-specific results are displayed in the table below. In the middle of the interface, two axes display method-specific figures after calculation. The panel below the axes consists of three push buttons and a radar button. The button “Calculate” invokes the analysis of the input data with the pre-defined method. The button “Clear” clears the two main axes and by clicking the “Save”-button an UI opens, where the user may choose where to save results. By clicking the “Hold Plot” radar button the user may superimpose results of subsequent analyses. By toggling the “Record”-button, all main steps and commands done by the user are saved in a queue. Pressing the “Generate Script”-button automatically generates a

Matlab-script and prompts the user to enter a file name and saving directory. The recording queue can be deleted by pressing the “Clear Record”-button.

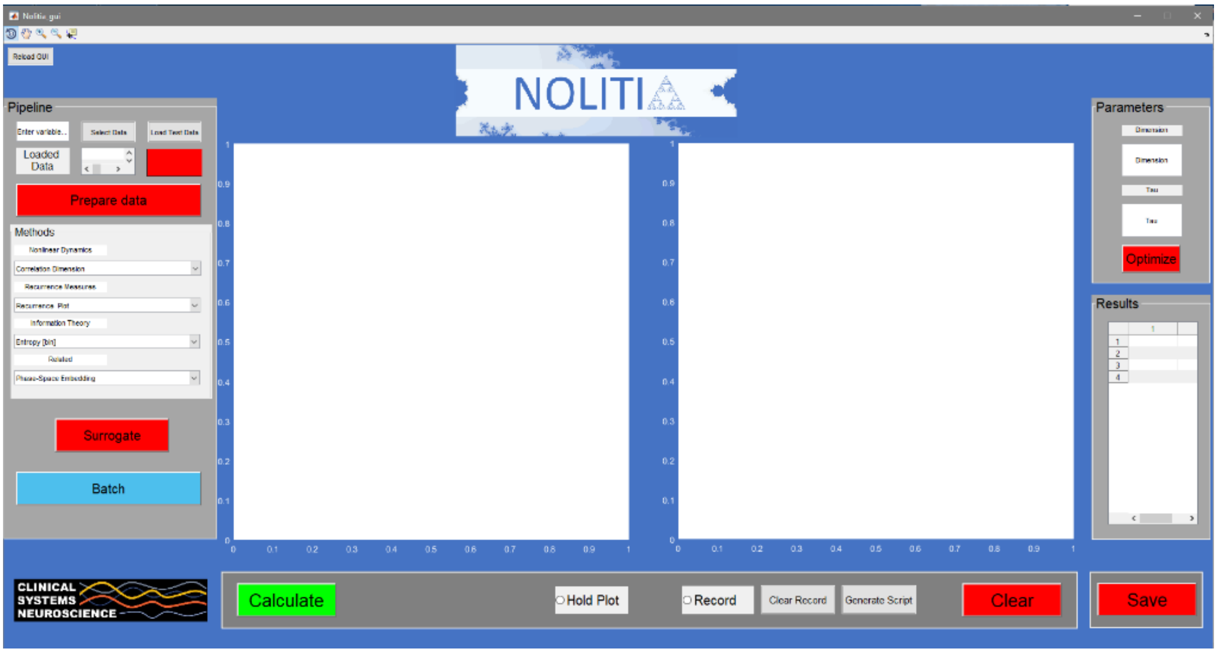

Figure 3: Graphical User Interface (GUI).

3.2.2. Workflow

Data Import:

The user may either choose to load a variable from workspace, from a “.mat-file” or to load some test data for training. Data must be an Nx1 vector for univariate methods or an Nx2 vector for bivariate methods like mutual information. The GUI only supports analysis of one dataset at a time. For stacked

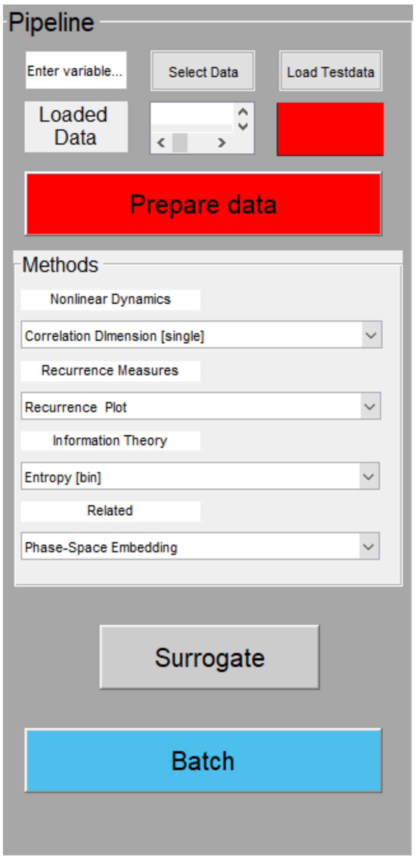

Figure 4: Pipeline of the GUI (left side).

analyses of multiple datasets, use the batch-editor (see section 2.3). Upon successful loading, the variable name is displayed in the field below "Select Data" and the red rectangle turns green. By clicking the "Surrogate"-Button the user may optionally transform the imported data into surrogate data using one of five different algorithms (see section 3, Table 59). After clicking, the button turns green indicating successful transformation. After clicking again on the button, the original data get reloaded, indicated by switching color from green to red.

Prepare Data:

The next optional step is to pre-process the input data. To begin, click on the red "prepare data" -button to invoke a table where pre-processing parameters may be specified. For a list of parameters, see Table 51. Note that for filtering options, the signal processing toolbox must be installed.

Select Method:

After successful specification, the button "Prepare Data" turns green and the user may choose an analysis method from one of the three dropdown-menus on the left side of the GUI.

Embedding Parameters:

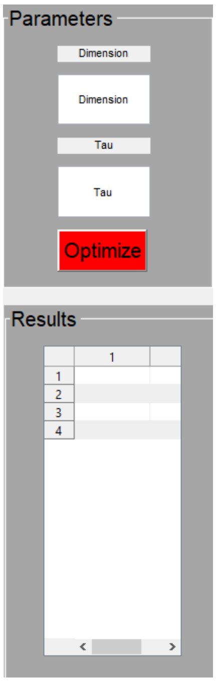

Depending on the chosen method, embedding parameters (dimension and tau) need to be specified to reconstruct the phase-space (see section 3: all functions with configuration structures requiring cfg.dim & cfg.tau). For this, the user may either choose to define both of them *ad-hoc* by typing them in the fields labeled "Dimension" and "Tau" on the right side of the GUI, or to optimize both using one of two different approaches by clicking the red "Optimize" button. For optimization parameters see section 4.4.4. After clicking "OK" the red "Optimize" -button turns green and optimum tau and dimension are displayed in the results table on the right side of the GUI. As optimized parameters are automatically defined, it is not necessary to enter them manually in the "Dimension" and "Tau" fields.

Figure 5: Embedding parameters and results table (right side)

Calculate, Save & Clear:

Finally, calculation can be invoked, by clicking the green "Calculate"-Button at the bottom of the GUI (Fig.6). Before calculation can proceed, the user is prompted to specify method-specific parameters (see section 3). Results are displayed in the table at the right of the GUI, while figures are presented in the two axes in the centre of the GUI. The left axis always displays the prepared time series. Results may be saved by clicking the red "Save"-button, while "Clear" clears the current axes and results table.

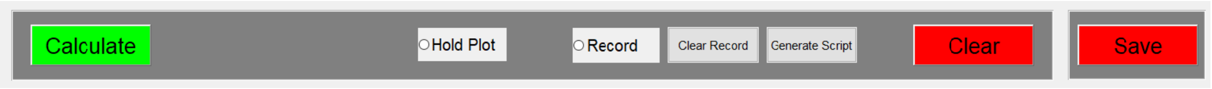

Figure 6: Plot, Clear and Save (bottom).

3.3. Batch-Editor

3.3.1. Overview

The batch-editor is intended to be a compromise between the accessibility of the GUI and the flexibility of custom-made scripts. In contrast to the GUI it allows for a semi-automatized stacked analysis of multiple datasets and methods. The batch-editor can be invoked by either clicking on the batch button in the GUI (Fig. 4) or by typing “batch_gui” in the command line of Matlab. The batch-editor is composed of three main parts. In the bottom half, the user loads datasets and chooses which data to analyse. In the top half, the user chooses which methods to use for analysis. In the center, linearly arranged buttons guide the user through the analysis pipeline (Fig. 7).

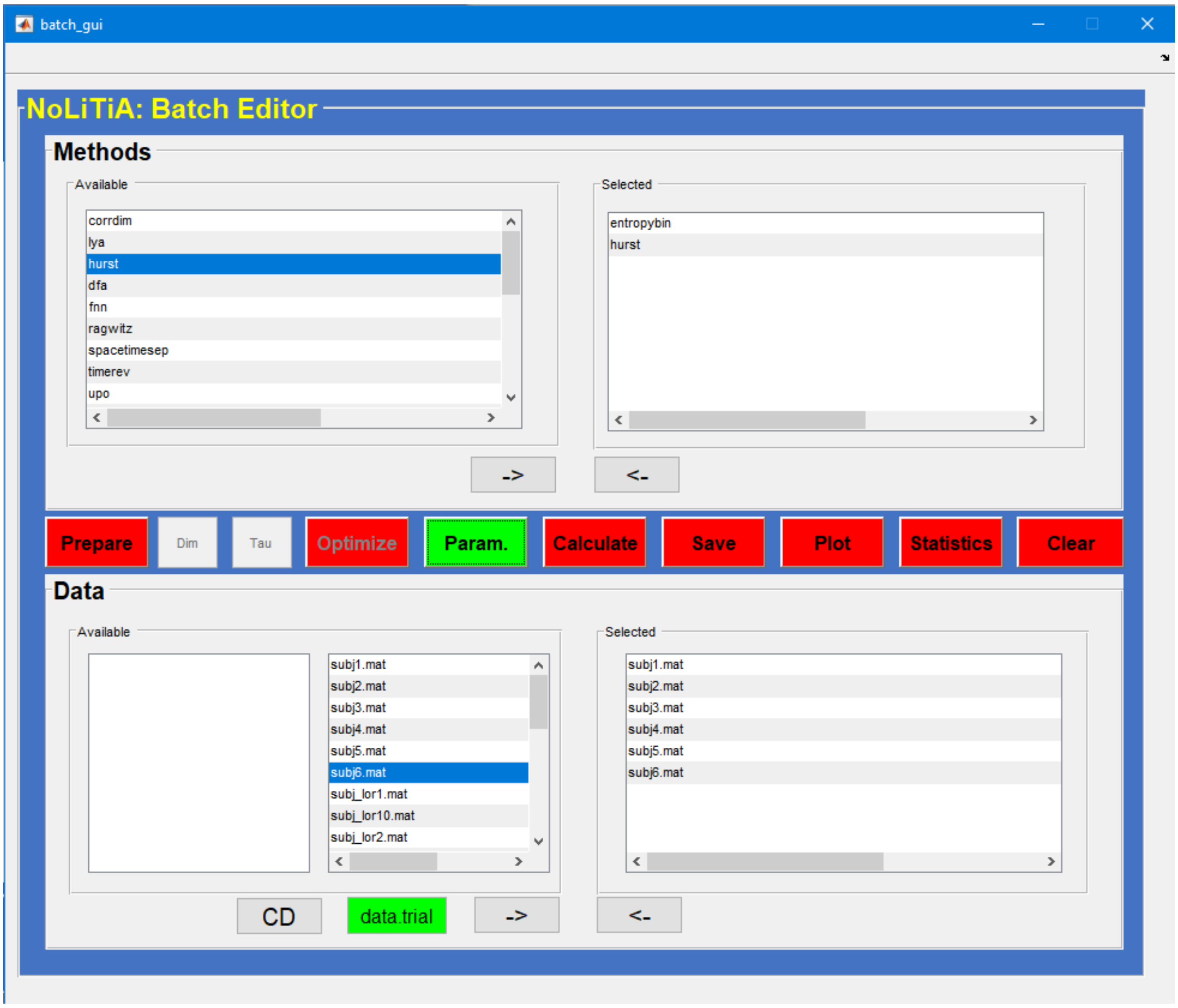

Figure 7: Batch-Editor.

3.3.2. Workflow

Data Import

The bottom part of the editor is dedicated to data import. The left table displays the current directory, while the center table shows the content of the current directory. To change the current directory either click on the dots at the top of the left table or click the CD-button. Before loading data, the user must specify a regular expression common to all datasets, in the red field next to the CD-button. For example, if the data to be analysed is saved in the field “trials” in the struct “data” the user enters: data.trials and hits enter. Upon successful specification, the field turns green. Next, the user sequentially chooses datasets by highlighting files with a left mouse-click and clicking on the right-pointing arrow at the bottom. Successfully loaded datasets appear on the right table. To unselect data, the user highlights the files in the right table and clicks the arrow pointing to the left. Data must be organized in cell arrays. Each cell may contain an n x m matrix, with n being time points and m being the observables/variables. In the context of neuroscience research, cells may represent trials and the matrices time x channel data of i.e. electrophysiological recordings.

Prepare Data

The next optional step is to pre-process the input data. To begin, the user clicks on the red “prepare” -button to invoke a table where pre-processing parameters may be specified. For a list with parameters, see Table 53. Note that for filtering options, the signal processing toolbox must be installed. The button turns green after pre-processing is finished.

Select Methods

The batch-editor allows for multiple analysis methods to be computed sequentially in an automatized fashion. To select a method, left-click on it in the top-left table and push the button with the right-pointing arrow. Upon successful selection, the method should appear in the top right table. To unselect a method, highlight it with a left-click and push the button with the arrow pointing to the left.

Embedding Parameters

Depending on the chosen methods, embedding parameters (dimension and tau) need to be specified to reconstruct the phase-space (see section 3: all functions with configuration structures requiring cfg.dim & cfg.tau). For this, the user may either choose to define both of them *ad-hoc* by typing them in the fields labeled “Dim” and “Tau”, or to optimize both for each dataset using one of two different approaches by clicking the red “Optimize” button. For optimization parameters see Table 51. The “Optimize”-Button turns green after the optimization procedure is finished. Note that optimization should not be applied if the user wants to plot time resolved topographic plots using the “plotting tool” (see section 3.4). Buttons and fields for embedding parameters are non-operable by default and become usable when selecting methods which necessitate phase-space reconstruction.

Define Parameters

By clicking on the “Param.”-button, the user is sequentially prompted to specify method-specific parameters (see section 3). The button turns green after parameter specification is complete.

Calculation, Saving and Clearing

Calculation can be invoked by clicking the “Calculate”-button. After successful calculation the button turns green and results may be saved by clicking the “Save”-button. Clicking on the “Clear”-Button reloads the batch-editor.

3.4. Plotting Tool

3.4.1. Workflow

Either clicking on the “Plot”-button or typing “plot_batch_gui” in the command line loads the “Plotting-Tool”. The plotting-tool is intended to be used for displaying results generated by the batch-editor. If the tool is loaded by clicking the “Plot”-button after computation in the batch-editor, the results of the first data set are automatically loaded into the plotting-tool. Alternatively, the user may load saved data by clicking the “Load”-button. Cell indices and matrix columns (observables) are displayed in the tables “Trials” and “Channel”, respectively. Available methods are represented by a hierarchical tree structure, which can be unfolded by clicking on “Methods” in the method panel. To display results,

left-click on a trial ID, channel ID, as well as on a method and push the “Plot”-button. Methods, which can be plotted using the “Plot”-button are indicated by a green arrow next to its name. Results are plotted in the center axis. By clicking the “Hold Plot” radar button the user may superimpose results of different datasets.

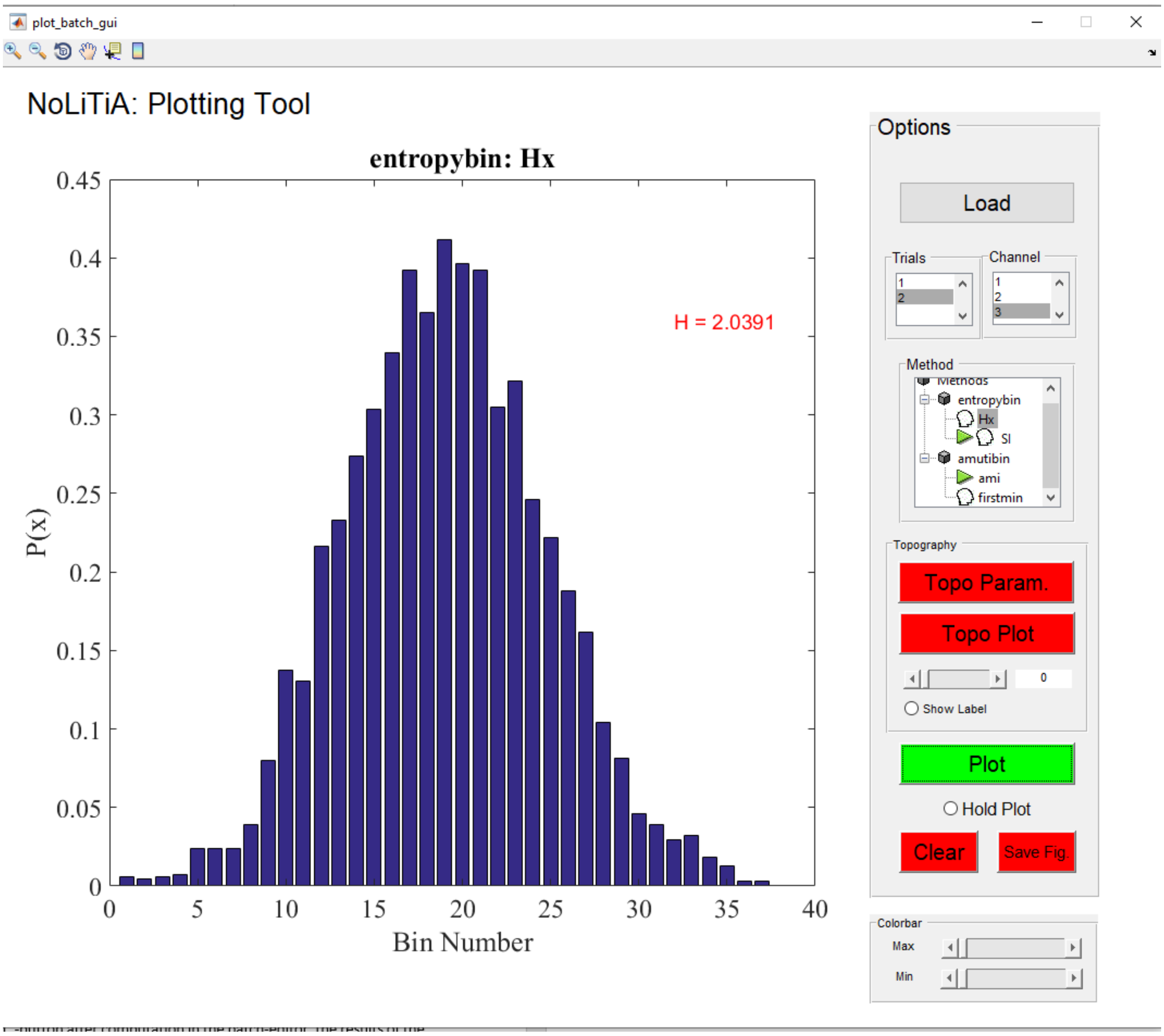

Figure 8: Plotting Tool.

3.4.2. Topographic Representation of EEG or MEG-Data

The plotting tool may be used to analyse electroencephalographic (EEG)- or magnetoencephalographic (MEG)-data, as each time series may represent one recorded channel. The plotting-tool allows for a

topographical representation of results per channel. By clicking on the red “Topo Param.”-button the user is prompted to select an “.sfp”-file containing the channel names and electrode positions. Additionally, the user may specify specific electrodes (1xnumelec) to plot, as well as a sampling frequency. Finally, after selecting a method from the list, the topographic plot is displayed after clicking the “Topo Plot”-button. Methods, which can be plotted using the “Topo Plot” button are indicated by a head shape next to its name. Colorbar limits can be adjusted by using the slider bar below the options panel. Methods with a green arrow, as well as a head shape symbol next to it may be plotted time resolved, by first clicking the “Topo Plot”-button and then using the slider below to scroll forward or backward in time. Supported function are listed in Table 1. For further function reference see section 3.

Table 1: Supported functions for Topo Plot

**Function**	**Field**	**Description**
**corrdim**	Dtakens	estimate of correlation dimension
**lya**	lle	estimate of maximum Lyapunov exponent
**ragwitz**	dimopt	estimate of Markov chain order
**ragwitz**	tauopt	estimate of embedding delay
**fnn**	firstmin	estimate of optimal embedding dimension
**timerev**	Qt	Time reversibility score
**timerev**	zstat	teststatistic for nonlinearity
**timerev**	sig	H0 of linearity rejected
**hurst**	expo	estimate of Hurst exponent
**dfa**	expo	estimate of self-affinity
**recurrenceplot**	rpde	recurrence period density entropy
**recurrenceplot**	det	determinism

**recurrenceplot**	lam	laminarity
**recurrenceplot**	rr	recurrence rate
**entropybin**	Hx	estimate of Shannon entropy
**entropybin**	SI	estimate of Shannon information
**entropykozachenko**	Hx	estimate of Shannon entropy
**entropykozachenko**	SI	estimate of Shannon information
**amutibin**	firstmin	first minimum of auto-mutual information
**amutiembknn**	firstmin	first minimum of auto-mutual information
**AIS**	AIS	active information storage
**AIS**	lAIS	local active information storage

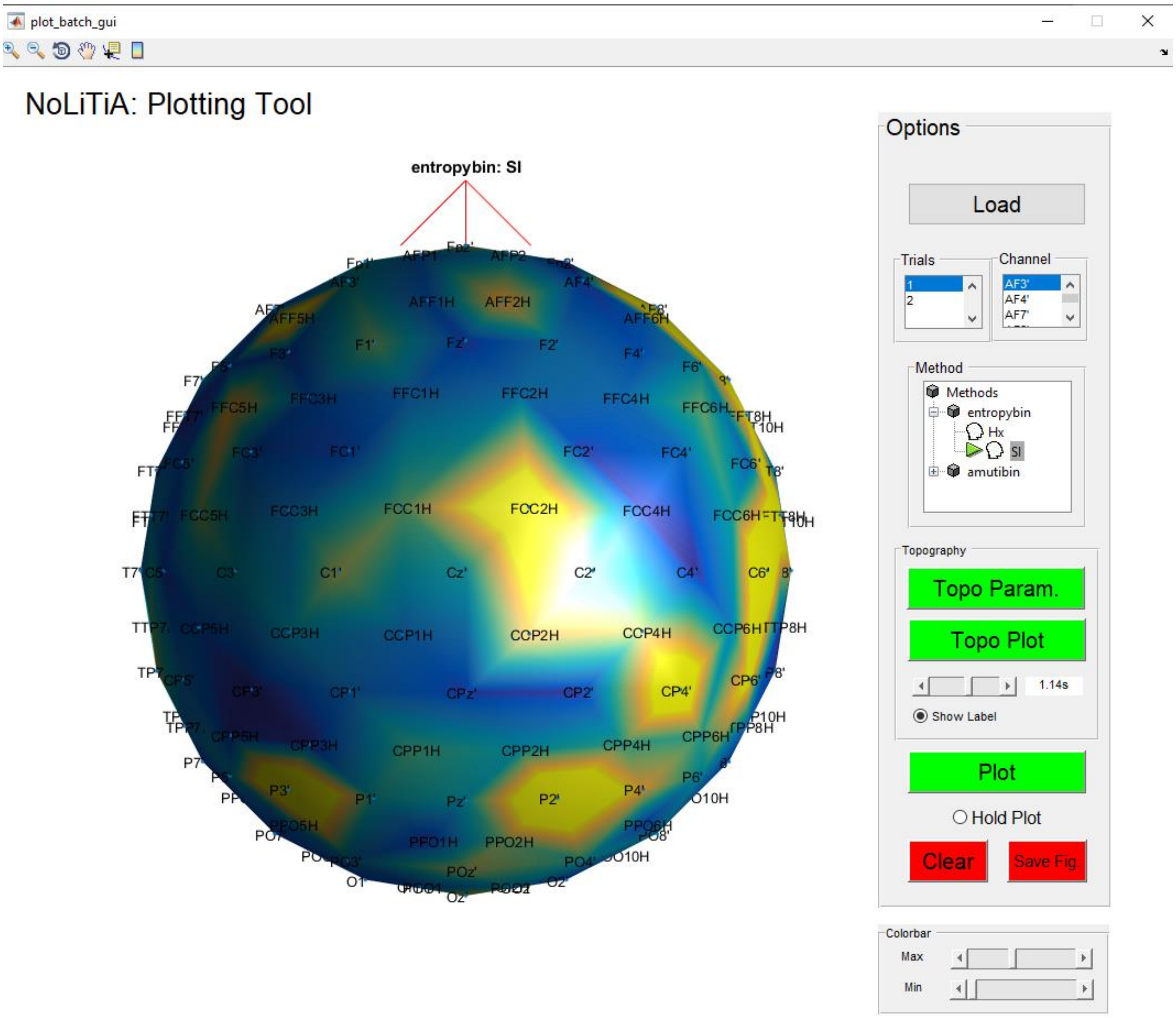

Figure 9: Topographic plot.

3.5. Statistics GUI

By clicking the “Statistics” button in the batch-editor or typing “nolitia_statistics_gui” in the command line it is possible to test statistical differences of estimated values between provided datasets. The “CD”-Button opens a pop-up window where the result-files of the batch computation may be selected. Pressing the arrow buttons (→,←) on the interface moves the highlighted files either to group 1 or group 2 which e.g., may represent different recording conditions. By clicking on any file in group 1 the method table on the left will display the available methods present in the data structure. Highlighting any of the methods will show the available variables in the variable table, which may be selected by

clicking on it. After choosing either “Monte-Carlo test” or “Cluster-based permutation analysis” (Maris and Oostenveld 2007) the computation may be initiated by clicking the “Compute” button. Results of the computation, including the p-values and whether or not the differences of datasets is significant, will be displayed in the “Output” table at the bottom. Results may be saved by clicking the “Save” button.

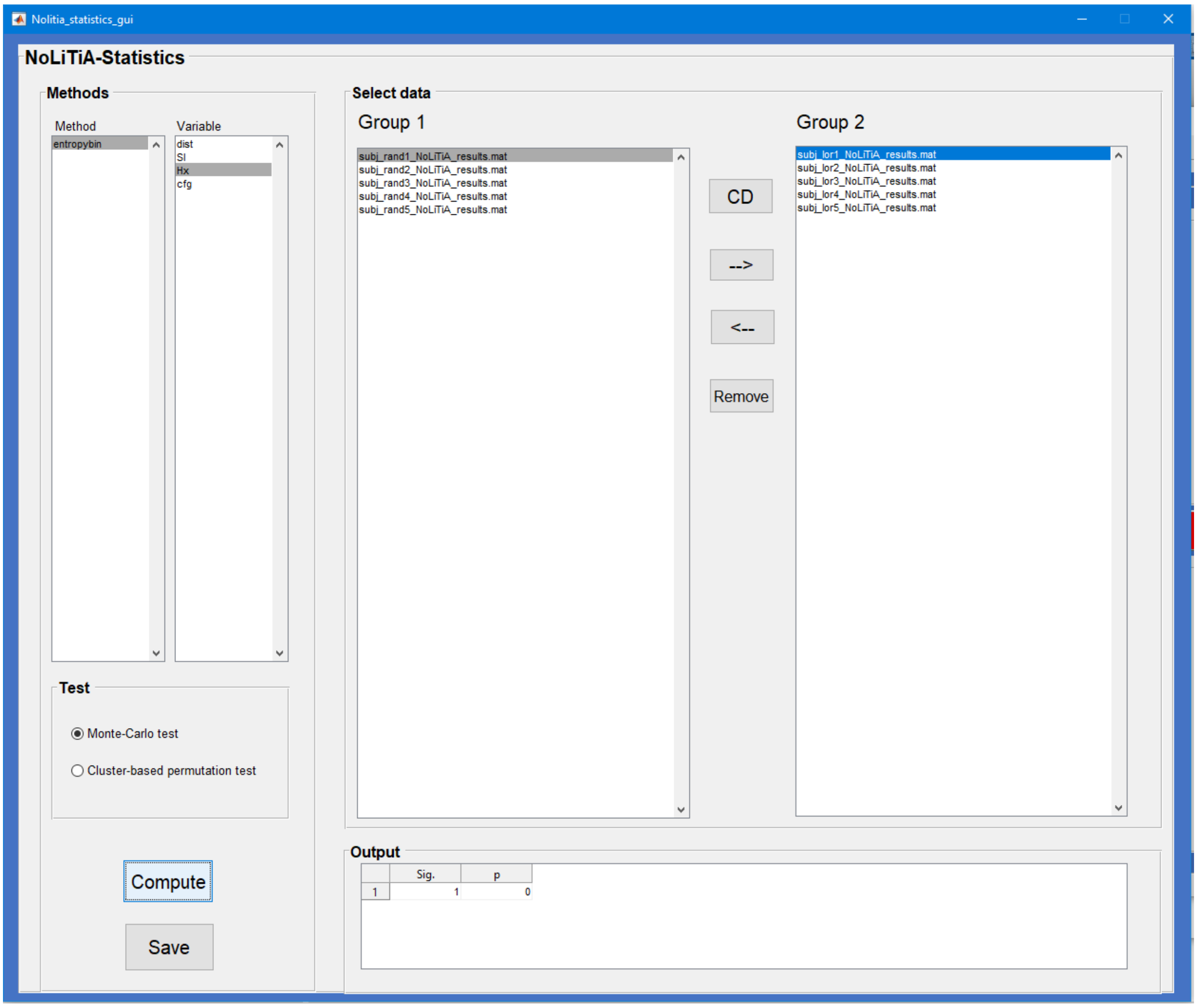

Figure 10: GUI of the statistics tool.

3.6. Custom-made Scripts

3.6.1. Overview

The final option to analyse data is by using custom-made scripts. This option offers the highest flexibility but requires some experience in Matlab-scripting. All core functions are built in the same way. Following the naming convention of other matlab toolboxes like Fieldtrip (Oostenveld et al. 2011) or SPM (The Wellcome Dept. of Imaging Neuroscience, London; www.fil.ion.ucl.ac.uk/spm), NoLiTiA functions all start with the prefix "nta". The user must provide up to two datasets (depending on whether the method is uni-or bivariate) and a configuration structure "cfg" containing method-specific parameters. Default values exist for all parameters. For a complete list of parameters per method see section 3. For beginners the GUI offers the possibility to record processing steps made in the GUI and to save them as a Matlab script (see section 3.2.1).

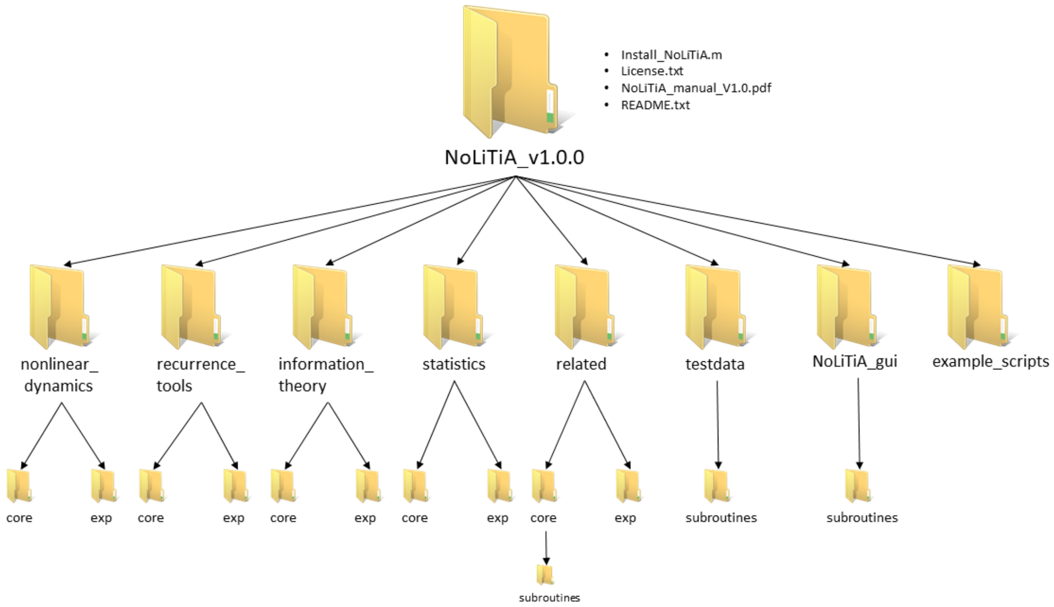

Figure 10: Folder structure. The main analysis functions are located in the „core"-folders. Content of the "exp"-folders is still under development and will be added to the core folder in future releases.

3.6.2. Workflow

The workflow is the same as previously described for the GUI and batch-editor. An example workflow is shown in figure 12.

Hint: Command line output can be supressed by setting the cfg.verbose parameter to zero.

```
%% Load data %%%%%%%%%%%%%%%%%%%%%%%%%%%%%%%%%%%%%%%%%%%%%%%%%%%%%%%%%%%%%%%%%%%%%%%

load('lorenz10000.mat')
data1           =   x;
data2           =   y;
results_final   =   [];   %define output structure

%% Prepare data %%%%%%%%%%%%%%%%%%%%%%%%%%%%%%%%%%%%%%%%%%%%%%%%%%%%%%%%%%%%%%%%%%%%

cfg             =   [];
cfg.normalize   =   1;
cfg.detrend     =   1;
cfg.filter      =   1;
cfg.lpfreq      =   100;
cfg.hpfreq      =   1;
cfg.fs          =   500;
cfg.toi         =   1:5000;
[prepared_data1]                    =   nta_prepare_data(data1,cfg);
[prepared_data2]                    =   nta_prepare_data(data2,cfg);

%% Optimize embedding parameters %%%%%%%%%%%%%%%%%%%%%%%%%%%%%%%%%%%%%%%%%%%%%%%%%%%

cfg             =   [];
cfg.optimization=   'deterministic';
cfg.dims        =   [2 9];
cfg.numbin      =   0; %Optimize bin size
[results_opt_emb]                   =   nta_optimize_embedding(prepared_data1,cfg);
results_final.opt_emb               =   results_opt_emb;

%% Analyze data %%%%%%%%%%%%%%%%%%%%%%%%%%%%%%%%%%%%%%%%%%%%%%%%%%%%%%%%%%%%%%%%%%%%

cfg             =   [];
cfg.minlength   =   0;
cfg.dim         =   results_opt_emb.optdim;
cfg.tau         =   results_opt_emb.opttau;
cfg.plt         =   0;
[results_rec]                       =   nta_recurrenceplot(prepared_data1,cfg);
results_final.rec                   =   results_rec;

%%%%%%%%%%%%%%%%%%%%%%%%%%%%%%%%%%%%%%%%%%%%%%%%%%%%%%%%%%%%%%%%%%%%%%%%%%%%%%%%%%%%
```

```
cfg             =   [];
cfg.numbin      =   0;
[results_MIbin]                    =   MIbin(prepared_data1,prepared_data2,cfg);
results_final.MIbin                =   results_MIbin;

%% Save data %%%%%%%%%%%%%%%%%%%%%%%%%%%%%%%%%%%%%%%%%%%%%%%%%%%%%%%%%%%%%%%%%

save('results_NoLiTiA.mat','results_final')
```

Figure 12: Example analysis pipeline.

4. Table of Functions

4.1. Nonlinear Dynamics Measures

4.1.1. Correlation Dimension

Function: *nta_corrdim.m*

Dependencies: *nta_autocorr.m, nta_amutibin.m, nta_phasespace.m*

Description: Calculation of the correlation sum C as a function of scales ε by which the correlation dimension D may be estimated (Grassberger and Procaccia 1983). Also calculates a maximum likelihood estimator for D by (Takens 1985):

$$C(\varepsilon) \sim \varepsilon^{D} \tag{1}$$

$$C(\varepsilon) = \frac{2}{N(N-1)} \sum_{i=1}^{N} \sum_{j=i+1}^{N} \Theta\left(\varepsilon - \left\| \mathbf{x}_i - \mathbf{x}_j \right\|\right) \tag{2}$$

$$D_{Takens} = \frac{C(\varepsilon_0)}{\int_0^{\varepsilon_0} C(\varepsilon)/\varepsilon \, d\varepsilon} \tag{3}$$

N: number of points, ||..||: distance norm, θ: Heaviside-step function.

Table 2: Configuration Structure (cfg)

Field Name	Description	Size	Data Type	Default

**tau**	embedding delay. If tau=0, the first minimum of the auto mutual information is used.	1x1	int	0
**dim**	embedding dimension	1x1	int	2
**ens**	min & max size of neighbourhood (% of maximal attractor diameter)	1x2	double	[1 100]
**nr**	number of neighbourhoods	1x1	int	10
**th**	Theiler window in samples. If th=0, 2*autocorrelation time is used.	1x1	int	0
**manual**	manually define the linear scaling region by choosing two points when the crosshair appears. If manual=0 logE(1:length(logE)/3) is used for slope calculation.	1x1	int	0
**resl**	number of points used for slope calculation	1x1	int	2
**plt**	plot results yes/no [1/0]	1x1	int	0
**verbose**	verbose level [1/0]	1x1	int	1

Table 3: Output Structure (results)

**Field Name**	**Description**	**Size**
**cfg**	configuration structure	struct
**logE_logC**	1. Column: log E (neighbourhood-size)<br>2. Column: log C (correlation sum)	nr x 2
**diff_logC**	local slope of logC per logE	1xlength(1:resl:nr)-1

**Dtakens**	estimate of the correlation dimension using Taken's estimator	1x1
**neighsizelist**	vector of neighbourhood-sizes used for calculation	1 x nr

4.1.2. Lyapunov Exponent

Function: *nta_lya.m*

Dependencies: *nta_autocorr.m, nta_amutibin.m, nta_phasespace.m, nta_neighsearch.mexw64*

Description: Estimation of the largest Lyapunov exponent λ as described in (Kantz 1994) (5) and (Rosenstein et al. 1993) (6):

$$|\mathbf{x}(\Delta t)| \approx e^{\lambda t}|\mathbf{x}(t_0)| \quad (4)$$

By plotting

$$S(\Delta t) = \frac{1}{N}\sum_{t_0=1}^{N} \ln\left(\frac{1}{|U(\mathbf{x}_{t_0})|}\sum_{\mathbf{x}_t \in U(\mathbf{x}_{t_0})} \left\|\mathbf{x}_{t_0+\Delta t} - \mathbf{x}_{t+\Delta t}\right\|\right) \quad (5)$$

$$S(\Delta t) = \frac{1}{N}\sum_{t_0=1}^{N} \ln\left(\left\|\mathbf{x}_{t_0+\Delta t} - \mathbf{x}_{t+\Delta t}\right\|\right), \quad (6)$$

as a function of temporal separation Δt, one can estimate λ by calculating the slope of a reasonable linear fit of the expansion rate S. N: Number of samples, U: neighbourhood, ||..||: distance norm.

Table 4: Configuration Structure (cfg)

Field Name	Description	Size	Data Type	Default
**tau**	embedding delay. If tau=0, the first minimum of the auto mutual information is used.	1x1	int	0
**dim**	embedding dimension	1x1	int	2
**method**	either choose Kantz ['Kantz'] or Rosenstein's ['Rosenstein'] algorithm	char	char	'Kantz'

**en**	size of neighbourhood (% of maximal attractor diameter)	1x1	double	5
**numran**	number of random points used for calculation	1x1	int	100
**it**	number of temporal iterations	1x1	int	10
**th**	Theiler window in samples. If th=0, 2*autocorrelation time is used.	1x1	int	0
**resl**	number of points used for slope estimation	1x1	int	2
**manual**	manually define the linear region by choosing two points when the crosshair appears. If manual=0 1:it/3 is used for slope calculation.	1x1	int	0
**plt**	plot results yes/no [1/0]	1x1	int	1
**verbose**	verbose level [1/0]	1x1	int	1

Table 5: Output Structure (results)

**Field Name**	**Description**	**Size**
**cfg**	configuration structure	struct
**lle**	estimate of the largest Lyapunov exponent	1x1
**loglya**	log of distances after it iterations	1 x it+1
**it**	vector with temporal iterations	1 x it+1
**diffloglya**	local slope of loglya per iteration	1 x length(1:resl:it)

**residuals**	residuals of line fitting	1 x (it/3)

4.1.3. Ragwitz Estimator

Function: *nta_ragwitz.m*

Dependencies: *nta_autocorr.m, nta_phasespace.m, nta_neighsearch.mexw64*

Description: Local constant predictor used for estimation of phase-space embedding parameters d and tau of Markov models (Ragwitz and Kantz 2002). Optimal choices for d and tau comprise combinations of both for which the root mean squared predication error is minimum:

$$\hat{\mathbf{x}}_{t+1}^{d_x}(d, tau) = \frac{1}{\left|U_\varepsilon(\mathbf{x}_t^{d_x})\right|} \sum_{\mathbf{x}_{t-\Delta t}^{d_x} \in U_\varepsilon(\mathbf{x}_t^{d_x})} \mathbf{x}_{t-\Delta t+1}^{d_x} \quad (7)$$

$$RMSPE(d, tau) = \sqrt{\frac{\sum_{t=1}^{N}\left(\hat{\mathbf{x}}_{t+\Delta t}^{d_x} - \mathbf{x}_{t+\Delta t}^{d_x}\right)^2}{N}} \quad (8)$$

U: local neighbourhood, ε: neighbourhood-size, N: number of points.

Table 6: Configuration Structure (cfg)

Field Name	Description	Size	Data Type	Default
**taus**	vector of tau to scan either in multiple of autocorrelation time in percent	1xN	int	[10:10:100]
**dims**	min. & max. embedding dimension	1x2	int	[2 9]
**mode**	how to define tau (taus). 'samples': taus in samples or 'multi'=taus in multiple of autocorrelation time in percent	char	char	'multi'
**mass**	number of neighbours per point	1x1	int	4

**hor**	prediction horizon	1x1	int	1
**metric**	distance norm 'euclidean' or 'maximum'	1x7 or 1x9	char	'maximum'
**plt**	plot results yes/no [1/0]	1x1	int	0
**verbose**	verbose level [1/0]	1x1	int	1

Table 7: Output Structure (results)

**Field Name**	**Description**	**Size**
**cfg**	configuration structure	struct
**tauopt**	optimum tau	1x1
**dimopt**	optimum dimension	1x1
**rmspe**	mean squared prediction error normalized to standard deviation of data	length(dims(1):dims(2)) x length(taus)
**prges**	first rows: predicted values, second rows: original values	2 x dims x taus

4.1.4. False Nearest Neighbours

Function: *nta_fnn.m*

Dependencies: *nta_amutibin.m, nta_phasespace.m*

Description: False Nearest Neighbours Algorithm by (Kennel et al. 1992). Calculates the number of false nearest neighbours fnn as a function of embedding dimensions d. False neighbours arise due to projections caused by an insufficient embedding dimension:

$$\mathrm{fnn}(d) = \sum \frac{\theta\left(\frac{|x_{i+1} - n(xi)_{j+1}|}{\|\mathbf{x}_i^d - \mathbf{n}_{xi}^d\|} - \mathrm{Rtol}\right)}{\wedge\ \theta\left(\sqrt{\left(\|\mathbf{x}_i^d - \mathbf{n}_{xi}^d\|\right)^2 - \left(|x_{i+1} - n(xi)_{j+1}|\right)^2} - \mathrm{STD}(X) * \mathrm{Atol}\right)} \quad (9)$$

n_x: next neighbour of x, ||..||: distance norm, θ: Heaviside-step function, Rtol: distance threshold, Atol: loneliness threshold, STD: standard deviation.

Table 8: Configuration Structure (cfg)

Field Name	Description	Size	Data Type	Default
**tau**	embedding delay. If tau=0, the first minimum of the auto mutual information is used.	1x1	int	0
**dim**	max embedding dimensions	1x1	int	9
**Rtol**	threshold parameter for distance	1x1	double	10
**Atol**	threshold parameter for loneliness	1x1	double	2
**thr**	threshold parameter for first minimum of false neighbours (%)	1x1	double	1
**plt**	plot results yes/no [1/0]	1x1	int	0
**verbose**	verbose level [1/0]	1x1	int	1

Table 9: Output Structure (results)

Field Name	Description	Size
**cfg**	configuration structure	struct

**fnn**	first row: % of false nearest neighbours, second row: tested dimensions	2 x length(dims(1):dims(2))
**firstmin**	dimension at first zero crossing	1x1

4.1.5. Space-Time Separation Plot

Function: *nta_spacetimesep.m*

Dependencies: *nta_amutibin.m, nta_phasespace.m, nta_neighsearch.mexw64*

Description: Space-Time-Separation-Plot. Can be used to determine Theiler-window (Provenzale et al. 1992). It shows the proportion of points closer than a distance ε at a given time separation Δt as a function of Δt:

$$\mathrm{Hto}(\Delta t) = p(||\mathbf{x}(t+\Delta t) - \mathbf{x}(t)|| < \varepsilon) \quad (10)$$

||..||: distance norm.

Table 10: Configuration Structure (cfg)

Field Name	Description	Size	Data Type	Default
**tau**	embedding delay	1x1	int	1
**dim**	embedding dimension	1x1	int	2
**nr**	Number of classes (succeeding classes contain an increasing proportion of points in phase-space)	1x1	int	5
**maxlag**	maximum number of temporal lags	1x1	int	100
**metric**	distance norm 'euclidean' or 'maximum'	1x7 or 1x9	char	'maximum'
**plt**	plot results yes/no [1/0]	1x1	int	1

**verbose**	verbose level [1/0]	1x1	int	1

Table 11: Output Structure (results)

Field Name	Description	Size
**cfg**	configuration structure	struct
**hto**	space-time-matrix: matrix containing proportions of state-space vector with a given distance at a specific time lag	nr x maxlag
**lagvector**	vector containing temporal lags	1 x maxlag
**classvector**	vector containing histogram intervals of distances	1 x nr

4.1.6. Time Inversion

Function: *nta_timerev.m*

Dependencies: *nta_surrogates.m*

Description: Test for time reversibility. In conjunction with appropriate surrogates, timerev can be used to test for nonlinearity (H0 = linear dynamics, alpha=0.05) (Schreiber and Schmitz 1997).

$$\mathrm{Q_t} = \langle (\mathrm{x_t} - \mathrm{x_{t-1}})^3 \rangle \qquad (11)$$

<..>: mean

Table 12: Configuration Structure (cfg)

Field Name	Description	Size	Data Type	Default
**lag**	delay	1x1	int	1

**numsurr**	number of surrogates	1x1	int	100
**surrmode**	mode==1: random shuffling<br>mode==2: phase randomization<br>mode==3: amplitude adjusted phase randomization<br>mode==4: cut time series at random point and flip second half<br>mode==5: cut time series at random point and switch halves	1x1	int	2
**numit**	Number of iterations for amplitude adjustement (mode==3)	1x1	int	10
**verbose**	verbose level [1/0]	1x1	int	1

Table 13: Output Structure (results)

**Field Name**	**Description**	**Size**
**cfg**	configuration structure	struct
**Qt**	time reversibility statistic	1x1
**zstat**	z-score of surrogate test	1x1
**sig**	H0 rejected [1] or not [0]	1x1

4.1.7. Hurst Exponent

Function: *nta_hurst.m*

Dependencies: -

Description: Estimation of Hurst exponent H as a measure for self-affinity of a time series (Hurst 1951):

$$\frac{\mathrm{R(n)}}{\mathrm{STD(n)}} = \mathrm{C} * \mathrm{n}^{\mathrm{H}} \tag{12}$$

$$\mathrm{R(n)} = \max\left(\sum_{i=1}^{t}(x_t - \bar{x})_i\right)_t - \min\left(\sum_{i=1}^{t}(x_t - \bar{x})_i\right)_t, \qquad t = 1..n \tag{13}$$

STD: standard deviation, n: window size, $\bar{x}$: average.

Table 14: Configuration Structure (cfg)

Field Name	Description	Size	Data Type	Default
**scale**	max. number of windows	1x1	int	10
**plt**	plot results yes/no [1/0]	1x1	int	1
**verbose**	verbose level [1/0]	1x1	int	1

Table 15: Output Structure (results)

Field Name	Description	Size
**cfg**	configuration structure	struct
**expo**	estimate of the exponent	1x1
**lognges**	log of window sizes	1 x scale
**logmeanrr**	log of rescaled ranges	1 x scale
**residuals**	residuals of line fitting	1 x scale
**meanresiduals**	mean of residuals	1x1

4.1.8. Detrended Fluctuation Analysis

Function: *nta_dfa.m*

Dependencies: -

Description: Calculation of detrended fluctuation analysis (DFA) to detect long-range correlations i.e. self-affinity, similar to the Hurst exponent H (Peng et al. 1994). In contrast to H DFA, can also be applied to nonstationary data:

$$X_t = \sum_{i=1}^{t} x_i - \bar{x} \quad (14)$$

$$F(n) = \sqrt{\frac{1}{N}\sum_{t=1}^{N}(X_t - Y_t)^2} \quad (15)$$

$$F(n) \propto n^a \quad (16)$$

X_t: unbounded process, $\bar{x}$: mean of x, n: window size, N: number of windows, Y_t: local linear fit of each window, a: scaling exponent.

Table 16: Configuration Structure (cfg)

Field Name	Description	Size	Data Type	Default
**scales**	min & max number of windows	1x2	int	[2 10]
**plt**	plot results yes/no [1/0]	1x1	int	1
**verbose**	verbose level [1/0]	1x1	int	1

Table 17: Output Structure (results)

Field Name	Description	Size
**cfg**	configuration structure	struct
**expo**	estimate of the exponent	1x1
**logbox**	log of window sizes	1 x length(scales(1):scales(2))
**logF**	log of temporal fluctuations	1 x length(scales(1):scales(2))
**residuals**	residuals of line fitting	1 x length(scales(1):scales(2))

**meanresiduals**	mean of residuals	1x1

4.1.9. Unstable Periodic Orbit Transform

Function: *nta_upo.m*

Dependencies: *nta_amutibin.m, nta_phasespace.m, nta_surrogates.m*

Description: Function to detect unstable periodic orbits in 2-dimensional phase-space (So et al. 1996). The algorithm concentrates points on nearby unstable period-one fixpoints. Significance of concentrated fixpoints may be tested by performing a surrogate test.

$$\hat{\boldsymbol{z}}_n = (\boldsymbol{1} - \boldsymbol{S}_n)^{-1}(\boldsymbol{z}_{n+1} - \boldsymbol{S}_n \boldsymbol{z}_n) \quad (17)$$

$$\boldsymbol{S}_n = \begin{pmatrix} a_n^1 & \cdots & a_n^d \\ & \boldsymbol{1} & \boldsymbol{0} \end{pmatrix} + \kappa \boldsymbol{R} \|\boldsymbol{z}_{n+1} - \boldsymbol{z}_n\| \quad (18)$$

$$\begin{pmatrix} a_n^1 \\ \vdots \\ a_n^d \end{pmatrix} = \begin{pmatrix} (\boldsymbol{z}_n - \boldsymbol{z}_{n-1})^\dagger \\ \vdots \\ (\boldsymbol{z}_{n-(d-1)} - \boldsymbol{z}_{n-d})^\dagger \end{pmatrix}^{-1} \begin{pmatrix} z_{n+1}^1 - z_n^1 \\ \vdots \\ z_{n-(d-2)}^1 - z_{n-(d-1)}^1 \end{pmatrix} \quad (19)$$

z_n: nth phase-space vector, $\hat{\boldsymbol{z}}_n$: transformed phase-space vector, **1**: identity matrix, d: dimension, κ: diffusion parameter, R: d x d random matrix drawn from the uniform interval [-1 1], ||..|| distance norm, †: transpose, z^1_n: first element of nth phase-space vector.

Table 18: Configuration Structure (cfg)

Field Name	Description	Size	Data Type	Default

**tau**	embedding delay	1x1	int	1
**numit**	number of iterations for upo transform	1x1	int	200
**bins**	number of bins per dimension	1x2	int	[10 10]
**numsurr**	number of surrogates	1x1	int	0
**surrmode**	mode==1: random shuffling mode==2: phase randomization mode==3: amplitude adjusted phase randomization mode==4: cut time series at random point and flip second half mode==5: cut time series at random point and switch halves	1x1	int	2
**plt**	plot results yes/no [1/0]	1x1	int	1
**verbose**	verbose level [1/0]	1x1	int	1

Table 19: Output Structure (results)

**Field Name**	**Description**	**Size**
**cfg**	configuration structure	struct
**bincenters**	center coordinates of histogram bins	{1 x bins(1)}, {1 x bins(2)}
**countorig**	normalized histogram counts of original phase-space	bins(1) x bins(2)
**counttrans**	normalized histogram counts of transformed phase-space	bins(1) x bins(2)

**countsurr**	normalized histogram counts of surrogates	bins(1) x bins(2)
**statcount**	z-score map of transformed phase space	bins(1) x bins(2)
**histdiag**	zscores of center diagonal of original and transformed phase-space	bins(1) x 2

4.2. Recurrence Measures

4.2.1. Recurrence Plot

Function: *nta_recurrenceplot.m*

Dependencies: *nta_amutibin.m, nta_phasespace.m, nta_neighsearch.mexw64*

Description: Calculates a recurrence matrix M as well as different recurrence based quantities (Eckmann, J-P., S. Oliffson Kamphorst, and David Ruelle. 1987; Little et al. 2007; Marwan et al. 2002; Romano et al. 2005).

$$M_{t,t+\Delta t} = \Theta(\varepsilon - ||\mathbf{x}_t - \mathbf{x}_{t+\Delta t}||) \qquad (20)$$

$$RR = \frac{1}{N^2}\sum_{i,j=1}^{N} M(i,j) \qquad (21)$$

$$\mathrm{DET} = \frac{\sum_{l=\mathrm{minl}}^{N} l p(l)}{\sum_{l=1}^{N} l p(l)} \qquad (22)$$

$$\mathrm{LAM} = \frac{\sum_{v=\mathrm{minl}}^{N} v p(v)}{\sum_{v=1}^{N} v p(v)} \qquad (23)$$

$$T = (t + \Delta t - \Delta t^{\mathrm{exit}}) - (t + \Delta t^{\mathrm{enter}}) \qquad (24)$$

$$P(T) = \frac{R(T)}{\sum_{i=T_{\min}}^{T_{\max}} R(i)}, T = T_{\min} \dots T_{\max} \qquad (25)$$

$$R_*(T) = \frac{R(T)}{T_{\max} - (T-1)}, \qquad (26)$$

$$\mathrm{RPDE} = -(\log_2 T_{\max})^{-1} \sum_{t=1}^{T_{\max}} P(T) \log_2 P(T) \qquad (27)$$

Θ: Heaviside-step function, ||..||: distance norm, ε: neighbourhood-size, N: number of points, minl: minimum length of diagonal/vertical lines, p: frequency distribution of line segments, T: recurrence time, Δt^{enter}: sample difference between $\mathbf{x}_t$ reentering U_ε and $\mathbf{x}_{t+\Delta t}$, Δt^{exit}: difference in samples between $\mathbf{x}_t$ and $\mathbf{x}_t$ first leaving U_ε, R(T): histogram with bin size equal to 1 sample and a number of bins equal to the longest recurrence time T_{max} , $R(T)^*$: normalized recurrence histogram, P(T): recurrence period

probabilities as a function of recurrence times, RR: recurrence rate, DET: determinism, LAM: laminarity, RPDE: recurrence period density entropy.

Table 20: Configuration Structure (cfg)

Field Name	Description	Size	Data Type	Default
**tau**	embedding delay	1x1	int	0
**dim**	embedding dimension	1x1	int	2
**en**	neighbourhood-size in % std of data	1x1	double	0
**rr**	recurrence rate in % of total possible rr	1x1	double	5
**minlengthdet**	minimum length of diagonal lines used for determinism estimation	1x1	int	0
**minlengthlam**	minimum length of vertical lines used for laminarity estimation	1x1	int	0
**minmaxRecPD**	minimum and maximum recurrence periods. Maximum must be smaller than length(input)-(tau*dim*((dim-1)/dim)).	1x2	int	[0 0]
**singlenei**	use only nearest neighbour for calculation of recurrence periods yes/no [1/0]	1x1	int	1
**norm**	normalize recurrence frequencies by total possible number of recurrences per period bin yes/no [1/0]	1x1	int	0

**metric**	distance norm 'euclidean' or 'maximum'	1x7 or 1x9	char	'maximum'
**plt**	plot results yes/no [1/0]	1x1	int	1
**verbose**	verbose level [1/0]	1x1	int	1

Table 21: Output Structure (results)

**Field Name**	**Description**	**Size**
**cfg**	configuration structure	struct
**rr**	recurrence rate	1x1
**det**	determinism	1x1
**lam**	laminariy	1x1
**ratio**	ratio of determinism and rr	1x1
**sumdist**	thresholded recurrence matrix	NxN, N=size(embedding matrix,1)
**alldist**	untresholded recurrence matrix	NxN, N=size(embedding matrix,1)
**ht**	recurrence period probabilites as a function of periods	1 x length(minmaxRecPD(1):minmaxRecPD(2))
**gaco**	generalized autocorrelation as a function of lags	1xN, N=size(embedding matrix,1)
**rpde**	recurrence period density entropy	1x1

4.2.2. Joint Recurrence Plot

Function: *nta_jointrecurrenceplot.m*

Dependencies: *nta_recurrenceplot.m*

Description: Calculates a joint recurrence matrix and recurrence based measures (Romano et al. 2004; Little et al. 2007; Marwan et al. 2002; Romano et al. 2005). It is defined as the Hadamard product of the individual recurrence plots of multiple time series:

$$\mathrm{JR}_{t,t+\Delta t} = \Theta(\varepsilon - ||\mathbf{x}_t - \mathbf{x}_{t+\Delta t}||) * \Theta(\varepsilon - ||\mathbf{y}_t - \mathbf{y}_{t+\Delta t}||) \quad (28)$$

Θ: Heaviside-step function, ||..||: distance norm, ε: neighbourhood-size.

Table 22: Configuration Structure (cfg)

Field Name	Description	Size	Data Type	Default
**taus**	embedding delays	1x2	int	[0 0]
**dims**	embedding dimensions	1x2	int	[2 2]
**ens**	neighbourhood-sizes in % std of data	1x2	int	[0 0]
**rrs**	recurrence rates in % of total possible rr	1x2	int	[5 5]
**minmaxRecPD**	minimum and maximum recurrence periods. Maximum must be smaller than length(input)-(tau*dim*((dim-1)/dim)).	1x2	int	[0 0]
**minlengthdet**	minimum length of diagonal lines used for determinism estimation	1x1	int	0
**minlengthlam**	minimum length of vertical lines used for laminarity estimation	1x1	int	0
**singlenei**	use only nearest neighbour for calculation of recurrence periods yes/no [1/0]	1x1	int	1
**norm**	normalize recurrence frequencies by total possible number of recurrences per period bin yes/no [1/0]	1x1	int	0

**metric**	distance norm 'euclidean' or 'maximum'	1x7 or 1x9	char	'maximum'
**plt**	plot results yes/no [1/0]	1x1	int	1
**verbose**	verbose level [1/0]	1x1	int	1

Table 23: Output Structure (results)

Field Name	Description	Size
**cfg**	configuration structure	struct
**rr**	recurrence rate	1x1
**det**	determinism	1x1
**lam**	laminariy	1x1
**ratio**	ratio of determinism and rr	1x1
**sumdist**	thresholded recurrence matrix	NxN, N=size(embedding matrix,1)
**alldist**	untresholded recurrence matrix	NxN, N=size(embedding matrix,1)
**ht**	recurrence period probabilites as a function of periods	1 x length(minmaxRecPD(1):minmaxRecPD(2))
**gaco**	generalized autocorrelation as a function of lags	1xN, N=size(embedding matrix,1)
**rpde**	recurrence period density entropy	1x1

4.2.3. Cross Recurrence Plot

Function: *nta_crossrecurrenceplot.m*

Dependencies: *nta_amutibin.m, nta_phasespace.m, nta_recurrenceplot.m*

Description: Calculates a cross recurrence matrix and recurrence based measures (Marwan and Kurths 2002; Little et al. 2007; Marwan et al. 2002; Romano et al. 2005). For generation of a cross recurrence plot, multiple time series are embedded into the same phase

space. A recurrence matrix is then calculated for the joint embedding space according to:

$$\mathrm{CR}_{t,t+\Delta t} = \Theta(\varepsilon - ||\mathbf{x}_t - \mathbf{y}_{t+\Delta t}||) \qquad (29)$$

Θ: Heaviside-step function, ||..||: distance norm, ε: neighbourhood-size.

Table 24: Configuration Structure (cfg)

Field Name	Description	Size	Data Type	Default
**tau**	embedding delay	1x1	int	1
**dim**	embedding dimension	1x1	int	2
**en**	neighbourhood-size in % std of data	1x1	double	0
**rr**	recurrence rate in % of total possible rr	1x1	double	5
**minmaxRecPD**	minimum and maximum recurrence periods. Maximum must be smaller than length(input)-(tau*dim*((dim-1)/dim)).	1x2	int	[0 0]
**minlengthdet**	minimum length of diagonal lines used for determinism estimation	1x1	int	0
**minlengthlam**	minimum length of vertical lines used for laminarity estimation	1x1	int	0
**singlenei**	use only nearest neighbour for calculation of recurrence periods yes/no [1/0]	1x1	int	1
**norm**	normalize recurrence frequencies by total possible number of recurrences per period bin yes/no [1/0]	1x1	int	0
**plt**	plot results yes/no [1/0]	1x1	int	1
**verbose**	verbose level [1/0]	1x1	int	1

Table 25: Output Structure (results)

Field Name	Description	Size
**cfg**	configuration structure	struct
**rr**	recurrence rate	1x1
**det**	determinism	1x1
**lam**	laminariy	1x1
**ratio**	ratio of determinism and rr	1x1
**sumdist**	thresholded recurrence matrix	NxN, N=size(embedding matrix,1)
**alldist**	untresholded recurrence matrix	NxN, N=size(embedding matrix,1)
**ht**	recurrence period probabilites as a function of periods	1x length(minmaxRecPD(1):minmaxRecPD(2))
**gaco**	generalized autocorrelation as a function of lags	1xN, N=size(embedding matrix,1)
**rpde**	recurrence period density entropy	1x1

4.2.4. Recurrence Frequencies_over range of neighbourhoods

Function: *nta_recfreq_en_scan.m*

Dependencies: *nta_recurrenceplot.m*

Description: Calculates recurrence frequencies as well as different recurrence based quantities over a range of neighbourhood-sizes (Little et al. 2007; Marwan et al. 2002; Romano et al. 2005; Eckmann, J-P., S. Oliffson Kamphorst, and David Ruelle. 1987).

$$P(T,\varepsilon) = \frac{R(T)}{\sum_{i=T_{min}}^{T_{max}} R(i)}, T = T_{min} \dots T_{max} \qquad (30)$$

T: recurrence time, ε: neighbourhood-size, R(T): histogram with bin size equal to 1 sample and a number of bins equal to the longest recurrence time T_{max} , P(T): recurrence period probabilities as a function of recurrence times.

Table 26: Configuration Structure (cfg)

Field Name	Description	Size	Data Type	Default
**tau**	embedding delay	1x1	int	0
**dim**	embedding dimension	1x1	int	2
**ens**	Neighbourhood-size in % std of data. Specify minimum size, step-size and maximum size.	1x3	double	[1 1 101]
**minmaxRecPD**	minimum and maximum recurrence periods. Maximum must be smaller than length(input)-(tau*dim*((dim-1)/dim))	1x2	int	[0 0]
**singlenei**	use only nearest neighbour for calculation of recurrence periods yes/no [1/0]	1x1	int	1
**norm**	normalize recurrence frequencies by total possible number of recurrences per period bin yes/no [1/0]	1x1	int	0
**metric**	distance norm 'euclidean' or 'maximum'	1x7 or 1x9	char	'maximum'
**plt**	plot results yes/no [1/0]	1x1	int	1
**verbose**	verbose level [1/0]	1x1	int	1

Table 27: Output Structure (results)

Field Name	Description	Size
**cfg**	configuration structure	struct
**rr**	recurrence rate	1x length([ens(1):ens(2):ens(3)])
**ht**	recurrence period probabilites as a function of periods	length([ens(1):ens(2):ens(3)]) x length(minmaxRecPD(1):minmaxRecPD(2))
**gaco**	generalized autocorrelation as a function of lags	length([ens(1):ens(2):ens(3)]) x N, N=size(embedding matrix,1)
**rpde**	recurrence period density entropy	1x length([ens(1):ens(2):ens(3)])

4.2.5. Windowed Recurrence Frequencies

Function: *nta_wind_recfreq.m*

Dependencies: *nta_recurrenceplot.m*

Description: Calculates windowed recurrence frequencies as well as other recurrence based quantities with an overlap of half a window (Little et al. 2007; Marwan et al. 2002; Romano et al. 2005; Eckmann, J-P., S. Oliffson Kamphorst, and David Ruelle. 1987).

$$P(T, w_n) = \frac{R(T, w_n)}{\sum_{i=T_{min}}^{T_{max}} R(i, w_n)}, T = T_{min} \dots T_{max} \quad (31)$$

T: recurrence time, w_n: nth temporal window of input time series x [x_n...$x_{windowsize}$], R(T): histogram with bin size equal to 1 sample and a number of bins equal to the longest recurrence time T_{max} , P(T): recurrence period probabilities as a function of recurrence times.

Table 28: Configuration Structure (cfg)

Field Name	Description	Size	Data Type	Default
**tau**	embedding delay	1x1	int	0
**dim**	embedding dimension	1x1	int	2
**en**	neighbourhood-size in % std of data	1x1	double	5
**window**	window size in samples.Parameter must be even number	1x1	int	1/10
**fs**	Sampling rate	1x1	int	-
**minlengthdet**	minimum length of diagonal lines used for determinism estimation	1x1	int	0
**minlengthlam**	minimum length of vertical lines used for laminarity estimation	1x1	int	0
**minmaxRecPD**	minimum and maximum recurrence periods. Maximum must be smaller than window-(tau*dim*((dim-1)/dim)).	1x2	int	[0 0]
**singlenei**	use only nearest neighbour for calculation of recurrence periods yes/no [1/0]	1x1	int	1
**norm**	normalize recurrence frequencies by total possible number of recurrences per period bin yes/no [1/0]	1x1	int	0
**metric**	distance norm 'euclidean' or 'maximum'	1x7 or 1x9	char	'maximum'
**plt**	plot results yes/no [1/0]	1x1	int	1
**verbose**	verbose level [1/0]	1x1	int	1

Table 29: Output Structure (results)

Field Name	Description	Size
**cfg**	configuration structure	struct
**time**	time vector in samples	time x 1
**resht**	recurrence period probabilites as a function of periods	length(minmaxRecPD(1):minmaxRecPD(2)) x time
**rr**	recurrence rate	1 x number of windows
**det**	determinism	1 x number of windows
**lam**	laminariy	1 x number of windows
**ratio**	ratio of determinism and rr	1 x number of windows
**gaco**	generalized autocorrelation as a function of lags	1xN, N=size(embedding matrix,1)
**rpde**	recurrence period density entropy	1 x number of windows

4.3. Information Theoretic Measures

4.3.1. Shannon Entropy (Bin Estimator)

Function: *nta_entropybin.m*

Dependencies: *nta_freeddiac.m*

Description: Calculates the Shannon entropy H and Shannon information SI using a binning estimator (Shannon CE 1949).

$$\mathrm{H_{bin}(X)} = -\sum_{\mathrm{x}} \mathrm{p_X(X = x)} \log_2 \mathrm{p_X(X = x)} \qquad (32)$$

$$\mathrm{SI_{bin}(x)} = -\log_2 p(x) \qquad (33)$$

P_x: probability of variable X taking the value x.

Table 30: Configuration Structure (cfg)

Field Name	Description	Size	Data Type	Default
**numbin**	number of bins. If numbin=0, the number of bins gets optimized according to the Freedman-Diaconis rule.	1x1	int	0
**verbose**	verbose level [1/0]	1x1	int	1

Table 31: Output Structure (results)

Field Name	Description	Size
**cfg**	configuration structure	struct
**Hx**	Shannon entropy [bit]	1x1
**SI**	Shannon information	1xN, N=length(input)
**dist**	Probability distribution	1 x numbin

4.3.2. Differential Entropy (Kozachenko‘s Estimator)

Function: *nta_entropykozachenko.m*

Dependencies: *nta_amutibin.m, nta_phasespace.m, nta_neighsearch.mexw64*

Description: Calculation of differential entropy H and local differential entropy SI using a nearest neighbour estimator (Kozachenko, L. F., and Nikolai N. Leonenko. 1987).

$$H_K(X) = -\psi(\mathrm{mass}) + \psi(N) + \frac{d}{N} * \sum_{i=1}^{N} \varepsilon(i) \qquad (34)$$

$$SI_K(x) = -\psi(mass) + \psi(N) + d * \varepsilon(x) \quad (35)$$

Ψ: digamma function, mass: number of nearest neighbours, N: total number of points, d: dimension, ε: neighbourhood-diameter.

Table 32: Configuration Structure (cfg)

Field Name	Description	Size	Data Type	Default
**tau**	embedding delay	1x1	int	1
**dim**	embedding dimension	1x1	int	2
**mass**	number of neighbours used for probability density estimation	1x1	int	4
**metric**	distance norm 'euclidean' or 'maximum'	1x7 or 1x9	char	'maximum'
**verbose**	verbose level [1/0]	1x1	int	1

Table 33: Output Structure (results)

Field Name	Description	Size
**cfg**	configuration structure	struct
**Hx**	Differential entropy [nats]	1x1
**SI**	Shannon information	1xN, N=length(input)

4.3.3. Mutual Information (Bin Estimator)

Function: *nta_MIbin.m*

Dependencies: *nta_freeddiac.m*

Description: Calculation of mutual information MI and local mutual information lMI between two signals using a binning estimator (Cover, Thomas M., and Joy A. Thomas. 1991).

$$\mathrm{MI_{bin}(X;Y) = H_{bin}(X) + H_{bin}(Y) - H_{bin}(X;Y)} \quad (36)$$

$$\mathrm{lMI_{bin}(x;y) = SI_{bin}(y) + SI_{bin}(y) - SI_{bin}(x;y)} \quad (37)$$

H: Shannon entropy.

Table 34: Configuration Structure (cfg)

Field Name	Description	Size	Data Type	Default
**numbin**	number of bins. If numbin=0, the number of bins gets optimized according to the Freedman-Diaconis rule.	1x1	int	0
**verbose**	verbose level [1/0]	1x1	int	1

Table 35: Output Structure (results)

Field Name	Description	Size
**cfg**	configuration structure	struct
**MI**	mutual information [bit]	1x1
**lMI**	Local mutual information	1xN, N=length(input)

4.3.4. Mutual Information (Kraskov‘s Estimator)

Function: *nta_MIkraskov.m*

Dependencies: *nta_amutibin.m, nta_phasespace.m, nta_neighsearch.mexw64*

Description: Calculation of mutual information and local mutual information between two signals using a nearest neighbour estimator (Kraskov et al. 2004).

$$MI_K(X,Y) = \psi(mass) + \psi(N) + \frac{\sum_{i=1}^{N} \psi(n_{xi}) + \psi(n_{yi})}{N} \quad (38)$$

$$lMI_K(x,y) = \psi(mass) + \psi(N) + \psi(n_x) + \psi(n_y) \quad (39)$$

Ψ: digamma function, mass: number of nearest neighbours, N: total number of points, n: number of neighbours.

Table 36: Configuration Structure (cfg)

Field Name	Description	Size	Data Type	Default
**taus**	embedding delays	1x2	int	[0 0]
**dims**	embedding dimensions	1x2	int	[2 2]
**mass**	number of nearest neighbours used for PDF estimation	1x1	int	4
**metric**	distance norm 'euclidean' or 'maximum'	1x7 or 1x9	char	'maximum'
**verbose**	verbose level [1/0]	1x1	int	1

Table 37: Output Structure (results)

Field Name	Description	Size
**cfg**	configuration structure	struct

**MI**	mutual information [nats]	1x1
**lMI**	Local mutual information	1xN, N=length(input)

4.3.5. Mutual Information Matrix (Bin & Kraskov)

Function: *nta_MImatrix.m*

Dependencies: *nta_MIkraskov.m, nta_MIbin.m*

Description: Column-wise calculation of mutual information using two estimators (Cover, Thomas M., and Joy A. Thomas. 1991; Kraskov et al. 2004). See 4.3.3 & 4.3.4 for details on algorithms.

Table 38: Configuration Structure (cfg)

Field Name	Description	Size	Data Type	Default
**mode**	estimator type: bin ['bin'] or nearest neighbour ['nn']	1x2 | 1/3	char	'bin'
**numbin**	number of bins. If numbin=0, the number of bins gets optimized according to the Freedman-Diaconis rule.	1x1	int	0
**taus**	embedding delays	1x2	int	[1 1]
**dims**	embedding dimensions	1x2	int	[2 2]
**mass**	number of nearest neighbours used for PDF estimation	1x1	int	4
**metric**	distance norm 'euclidean' or 'maximum'	1x7 or 1x9	char	'maximum'
**verbose**	verbose level [1/0]	1x1	int	1

Table 39: Output Structure (results)

Field Name	Description	Size
**cfg**	configuration structure	struct
**MIMaabs**	mutual information Matrix (absolute values)	numbin x numbin
**MIManorm**	mutual information Matrix (normalized)	numbin x numbin

4.3.6. Auto-Mutual Information (Bin Estimator)

Function: *nta_amutibin.m*

Dependencies: *nta_MIbin.m*

Description: Calculates the auto-mutual information as a function of lags using a binning estimator (Cover, Thomas M., and Joy A. Thomas. 1991).

$$\mathrm{AMI_{bin}}(\mathrm{X};\mathrm{X}+\Delta\mathrm{t}) = \mathrm{H_{bin}}(\mathrm{X}) + \mathrm{H_{bin}}(\mathrm{X}+\Delta\mathrm{t}) - \mathrm{H_{bin}}(\mathrm{X},\mathrm{X}+\Delta\mathrm{t}) \qquad (40)$$

H: Shannon entropy

Table 40: Configuration Structure (cfg)

Field Name	Description	Size	Data Type	Default
**numbin**	number of bins	1x1	int	10
**maxlag**	maximum number of lags	1x1	int	half data length
**plt**	plot results yes/no [1/0]	1x1	int	1
**verbose**	verbose level [1/0]	1x1	int	1

Table 41: Output Structure (results)

Field Name	Description	Size
**cfg**	configuration structure	struct

**ami**	auto mutual information as a function of time [bit]	1 x maxlag
**firstmin**	first minimum of auto-mutual information	1x1

4.3.7. Auto-Mutual Information (Kraskov's Estimator)

Function: *nta_amutiembknn.m*

Dependencies: *nta_MIdelayunimulti.m*

Description: Calculates the auto-mutual information as a function of lags Δt using a nearest neighbours estimator (Kraskov et al. 2004).

$$AMI_K(\mathrm{X}, \mathrm{X} + \Delta \mathrm{t}) = \psi(mass) + \psi(N) + \frac{\sum_{i=1}^{N} \psi(n_{xi}) + \psi(n_{\mathrm{X}+\Delta \mathrm{t}i})}{N} \quad (41)$$

Ψ: digamma function, mass: number of nearest neighbours, N: total number of points, n: number of neighbours.

Table 42: Configuration Structure (cfg)

Field Name	Description	Size	Data Type	Default
**tau**	embedding delay	1x1	int	1
**dim**	embedding dimension	1x1	int	2
**mass**	number of nearest neighbours used for PDF estimation	1x1	int	4
**maxlag**	maximum number of lags	1x1	int	half data length
**metric**	distance norm 'euclidean' or 'maximum'	1x7 or 1x9	char	'maximum'
**plt**	plot results yes/no [1/0]	1x1	int	1

**verbose**	verbose level [1/0]	1x1	int	1

Table 43: Output Structure (results)

Field Name	Description	Size
**cfg**	configuration structure	struct
**embAMI**	auto-Mutual information per time lag [nats]	1 x maxlag
**firstmin**	first minimum of embAMI	1x1
**AIS**	active information storage	1x1

4.3.8. Active Information Storage

Function: *nta_AIS.m*

Dependencies: *nta_MIdelayunimulti.m*

Description: Calculates the active information storage using a nearest neighbours estimator (Lizier et al. 2012). Active information storage is defined as the mutual information between the current time point t of a random variable and the next past state at t-1. It is a measure for the average past information currently in use at each time point :

$$AIS(X) = MI_K(\boldsymbol{X}_{t-1}^{d_x}, X_t), \quad (42)$$

MI: mutual information, d: embedding dimension.

Table 44: Configuration Structure (cfg)

Field Name	Description	Size	Data Type	Default
**tau**	embedding delay	1x1	int	1
**dim**	embedding dimension	1x1	int	2

**mass**	number of nearest neighbours used for PDF estimation	1x1	int	4
**metric**	distance norm 'euclidean' or 'maximum'	1x7 or 1x9	char	'maximum'
**verbose**	verbose level [1/0]	1x1	int	1

Table 45: Output Structure (results)

Field Name	Description	Size
**cfg**	configuration structure	struct
**AIS**	active information storage [nats]	1x1
**lAIS**	Local active information storage	1xN, N=length(input)

4.4. Related

4.4.1. Phase Space reconstruction

Function: *nta_phasespace.m*

Dependencies: *neighsearch.mexw64*

Description: Embedding of time series in phase-space using Taken's delay embedding (Takens 1981). By time-shifting a univariate time series d times by a factor τ, the time series can be embedded in a d-dimensional phase-space:

$$\boldsymbol{x}_t^{d_x} = [\mathrm{x}_{\mathrm{t}-(\mathrm{d}_x-1)\tau}, \mathrm{x}_{\mathrm{t}-(\mathrm{d}_x-2)\tau}, \dots, \mathrm{x}_{\mathrm{t}-\tau}, \mathrm{x}_{\mathrm{t}}]^T \quad (43)$$

T: transpose

The function also allows to perform two nonlinear noise reduction schemes: 1) a simple nonlinear noise reduction where each vector is replaced by a weighted average of its closest neighbours and 2)

a projection scheme, where the local neighbourhood gets projected onto a local manifold along the eigenvectors with the largest eigenvalues.

Table 46: Configuration Structure (cfg)

Field Name	Description	Size	Data Type	Default
**tau**	embedding delay	1x1	int	1
**dim**	embedding dimension	1x1	int	2
**filter**	mode of nonlinear noise reduction [none/simple/projection]		char	none
**en**	neighbourhood size for nonlinear noise reduction	1x1	double	0
**ensize**	determine whether neighbourhood-size are absolute or relative values in % of attractor diameter [rel/abs]		char	abs
**Q**	number of largest components to use for noise reduction	1x1	int	2
**keepfiltTS**	keep filtered univariate time series [1/0]	1x1	int	1
**keepresidual**	keep residual univariate time series [1/0]	1x1	int	1
**verbose**	verbose level [1/0]	1x1	int	1

Table 47: Output Structure (results)

Field Name	Description	Size
**cfg**	configuration structure	struct
**embTS**	phase-space coordinates	time x dim
**residual**	extracted noise	time x 1
**filtTS**	Filtered time series	time x 1

4.4.2. Autocorrelation

Function: *nta_autocorr.m*

Dependencies: *xcorr.m*

Description: Calculates the autocorrelation R as a function of delays τ:

$$\boldsymbol{R}(\boldsymbol{\tau}) = \sum_{t=0}^{N-\tau-1} x(t)x(t+\tau) \qquad (44)$$

N: length of time series, τ: time lag

Table 48: Configuration Structure (cfg)

Field Name	Description	Size	Data Type	Default
**lag**	number of temporal lags in samples	1x1	int	half data length

Table 49: Output Structure (results)

Field Name	Description	Size
**Rmm**	one-sided autocorrelation as a function of temporal lag in samples	1 x lag

4.4.3. Neighbours Distance (mex)

Function: *nta_neighsearch.mex64*

Dependencies: -

Description: Calculates a distance matrix of phase-space states using the Euclidean or maximum norm.

$$\mathrm{Dist} = ||\mathbf{x}_{\mathrm{t}} - \mathbf{x}_{\mathrm{t+\Delta t}}|| \qquad (45)$$

||..||: distance norm.

Table 50: Input

Inputs	Description	Size	Data Type	Default
**Embedding matrix**	Output of phasespace.m	samples x dim	double	-
**Qx1 vector**	Indices of query points	Qx1 vector	int	-

Table 51: Output Structure (results)

Output	Description	Size
**sumdist**	Distance matrix (Euclidean)	NxN, N=size(input,1)

4.4.4. Optimize Embedding Parameters

Function: *nta_optimize_embedding.m*

Dependencies: *nta_fnn.m, nta_amutibin.m, nta_ragwitz.m, nta_phasespace.m*

Description: This function optimizes embedding parameters (dimension and tau (Cao 1997; Ragwitz and Kantz 2002). See 4.1.3, 4.1.4 & 4.3.6 for details on algorithms.

Table 52: Configuration Structure (cfg)

Field Name	Optimization	Description	Size	Data Type	Default
**optimization**	-	choose optimization procedure 'deterministic' (false nearest neighbours (dimension) & auto-mutual information (tau))	char	char	deterministic'

		or 'markov' (Ragwitz estimator (dimension & tau))			
**dims**	deterministic	min and max embedding dimensions	1x2	int	[2 9]
**R**	deterministic	Threshold Parameter for initial distance	1x1	double	10
**numbin**	deterministic	number of bins	1x1	int	0
**dims**	markov	min and max embedding dimensions	1x2	int	[2 9]
**taus**	markov	vector of tau to scan either in multiple of autocorrelation time in percent	1xN	int	[10:10:100]
**mass**	markov	number of neighbours per point	1x1	int	4
**hor**	markov	prediction horizon	1x1	int	1
**mode**	markov	how to define tau (taus). 'samples': taus in samples or 'multi'=taus in multiple of autocorrelation time in percent	1x5 | 1x7	char	multi'
**embed**	-	embed input data yes/no [1/0]	1x1	int	0

Table 53: Output Structure (results)

Field Name	Description	Size
**cfg**	configuration structure	struct
**optdim**	optimized embedding dimension	1x1
**opttau**	optimized embedding delay	1x1

4.4.5. Optimize Number of Bins for Histogram

Function: *nta_freeddiac.m*

Description: Low-level function to optimize histogram bins (Freedman, David, and Persi Diaconis 1981).

$$\text{Number of bins} = \frac{\max(x) - \min(x)}{2 * \text{IQR} * \sqrt[3]{N}} \quad (46)$$

IQR: inter quartile range, N: total number of points.

4.4.6. Prepare Data

Function: *nta_prepare_data.m*

Description: Preprocesses data by normalization, trend correction and filtering.

Table 54: Configuration Structure (cfg)

Field Name	Description	Size	Data Type	Default
**toi**	time points of interest in samples	1xN	int	0
**normalize**	z-normalization yes/no [1/0]	1x1	int	1
**detrend**	remove linear trend yes/no [1/0]	1x1	int	0
**filter**	filter yes/no [1/0]	1x1	int	0

**lpfreq**	lowpass frequency in Hz	1x1	int	100
**hpfreq**	highpass frequency in Hz	1x1	int	1
**causal**	apply causal filter yes/no [1/0]	1x1	int	0
**fs**	Sampling frequency in Hz	1x1	int	1000

Table 55: Output Structure (results)

**Field Name**	**Description**	**Size**
**prep_data**	prepared data	time x 1

4.5. Data

4.5.1. Lorenz Data Generation

Function: *nta_lorenzgen.m*

Description: Generates x,y and z component of the Lorenz-system with typical parameters for chaotic dynamics (Lorenz 1963).

$$\dot{X} = a(Y - X)$$
$$\dot{Y} = X(b - Z) - Y \qquad (47)$$
$$\dot{Z} = XY - cZ$$

a=10, b=28, c=8/3

Table 56: Input

**Inputs**	**Description**	**Size**	**Data Type**	**Default**
**L**	time vector at which the Lorenz-function should be evaluated e.g. 1:0.01:1000	1x time	double	-

Table 57: Output

Output	Description	Size
**x**	x component of Lorenz system	time x 1
**y**	y component of Lorenz system	time x 1
**z**	z component of Lorenz system	time x 1

4.5.2. Sinusoid Generation

Function: *nta_signalgen.m*

Description: Generates superimposed sinusoids.

$$y(x) = \begin{pmatrix} a^1 \\ \vdots \\ a^n \end{pmatrix} * \begin{pmatrix} \sin(x + \phi^1) \\ \vdots \\ \sin(x + \phi^n) \end{pmatrix} + \eta \quad (48)$$

a^n: amplitude of nth sinusoid, ϕ^n: phase of nth sinusoid, η: uniform white noise.

Table 58: Input

Inputs	Description	Size	Data Type	Default
**signalinf**	amplitude, frequency, phase	nx3	double	-
**sec**	time length in seconds		double	-
**fs**	sampling frequency in Hz		double	-
**noise**	amplitude of added noise in % STD of sinusoid		double	-

Table 59: Output Structure (results)

Output	Description	Size
**y**	oscillatory signal	time x 1

4.5.3. Logistic Map

Function: *nta_logisticmap.m*

Description: This function computes the logistic map or "Verhulst equation".

$$f(x) = rx(1 - x) \tag{49}$$

r: control parameter.

Inputs	Description	Size	Data Type	Default
**numit**	number of iterations	1x1	int	-
**r**	control parameter (chaotic behavior @ r>3.57)	1x1	double	-

Output	Description	Size
**y**	iterated time series	numit+1 x 1

4.5.4. Lorenz Data

Mat-File: *lorenz5000.mat* (5000 samples of x-component of the Lorenz-function)

lorenz10000.mat (10000 samples of x,y,z-components of the Lorenz-function)

4.6. Statistics

4.6.1. Generation of Surrogates

Function: *nta_surrogates.m*

Dependencies: -

Description: Generates surrogate data using five different algorithms (Schreiber and Schmitz 1996).

Table 60: Configuration Structure (cfg)

Field Name	Description	Size	Data Type	Default
**mode**	mode==1 random shuffling<br>mode==2 phase randomization<br>mode==3 amplitude adjusted phase randomization<br>mode==4 cut time series at random point and flip second half<br>mode==5 cut time series at random point and switch halves	1x1	int	1
**numit**	Number of iterations for amplitude adjustment (mode==3)	1x1	int	0

Table 61: Output Structure (results)

Field Name	Description	Size
**cfg**	configuration structure	struct
**surr**	surrogate time series	length(input data) x 1

4.6.2. Monte-Carlo Test

Function: *nta_nolitia_monte.m*

Dependencies: -

Description: Tests statistical differences of provided datasets using nonparametric Monte-Carlo simulations.

Table 62: Input

Inputs	Description	Size	Data Type	Default
**data1**	input data (cell array of subjects)	1xN	cell array	-
**data2**	input data (cell array of subjects)	1xN	cell array	-

Table 63: Configuration Structure (cfg)

Field Name	Description	Size	Data Type	Default
**numperm**	Number of permutations	1x1	int	1000
**vars**	Variables to test	1xN	int	1
**outputvar**	Specififc output variable of vars to test	1xN	char	-
**alpha**	Alpha level to test	1x1	double	0.05
**bonf**	Bonferroni correction yes/no [1/0]	1x1	int	0
**verbose**	Verbose level [1/0]	1x1	int	1
**method**	Name of method to be tested	1xN	char	-

Table 64: Output Structure (results)

Field Name	Description	Size
**cfg**	configuration structure	struct
**P**	p-value	1x1
**sig**	Significant yes/no [1/0]	1x1
**diffsurr_grand**	grand mean of differences between surrogate datasets	1x1
**difforig_grand**	grand mean of differences between original datasets	1x1

4.6.3. Cluster-based permutation analysis

Function: *nta_nolitia_cbpa.m*

Dependencies: image processing toolbox (for fast calculations), connsets7 (alternative), nolitia_t_values

Description: Tests statistical differences of provided datasets using nonparametric cluster-based permutation analysis (Maris and Oostenveld 2007).

Table 65: Input

Inputs	Description	Size	Data Type	Default
**data1**	input data (cell array of subjects)	1xN	cell array	-
**data2**	input data (cell array of subjects)	1xN	cell array	-

Table 66: Configuration Structure (cfg)

Field Name	Description	Size	Data Type	Default
**numperm**	Number of permutations	1x1	int	1000
**vars**	Variables to test	1xN	int	1
**clustalpha**	Alpha level for clusters	1xN	char	0.05
**alpha**	Final alpha level to test	1x1	double	0.05
**maxcluster**	How to define clustervalue: sizec=maximum size of cluster, sumt= sum of t-values	1xN	char	'sizec'
**verbose**	Verbose level [1/0]	1x1	int	1
**method**	Name of method to be tested	1xN	char	-

Table 67: Output Structure (results)

Field Name	Description	Size

**cfg**	configuration structure	struct
**pos_cluster**	Statistics of positive clusters found	1xvars
**neg_cluster**	Statistics of negative clusters found	1xvars
**p**	p-value	1xnumber of clusters
**ids**	Indices of cluster samples	1xnumber of clusters
**sig**	Significant yes/no [1/0]	1xnumber of clusters
**averages**	Mean values of input data	nx2

Literature

Cao, Liangyue (1997): Practical method for determining the minimum embedding dimension of a scalar time series. In *Physica D: Nonlinear Phenomena* 110 (1-2), pp. 43–50. DOI: 10.1016/S0167-2789(97)00118-8.

Cover, Thomas M., and Joy A. Thomas. (1991): Elements of information theory.

Eckmann, J-P., S. Oliffson Kamphorst, and David Ruelle. (1987): Recurrence plots of dynamical systems. In *EPL (Europhysics Letters)* (973).

Freedman, David, and Persi Diaconis (1981): On the histogram as a density estimator: L 2 theory. In *Zeitschrift für Wahrscheinlichkeitstheorie und verwandte Gebiete*, 453-476.

Grassberger, Peter; Procaccia, Itamar (1983): Measuring the strangeness of strange attractors. In *Physica D: Nonlinear Phenomena* 9 (1), pp. 189–208. DOI: 10.1016/0167-2789(83)90298-1.

Hurst, H. E. (1951): Long-term storage capacity of reservoirs. In *Trans. Amer. Soc. Civil Eng.*, pp. 770–808.

Kantz, Holger (1994): A robust method to estimate the maximal Lyapunov exponent of a time series. In *Physics Letters A* 185 (1), pp. 77–87. DOI: 10.1016/0375-9601(94)90991-1.

Kennel, Matthew B.; Brown, Reggie; Abarbanel, Henry D. I. (1992): Determining embedding dimension for phase-space reconstruction using a geometrical construction. In *Phys. Rev. A* 45 (6), pp. 3403–3411. DOI: 10.1103/PhysRevA.45.3403.

Kozachenko, L. F., and Nikolai N. Leonenko. (1987): Sample estimate of the entropy of a random vector. In *Problemy Peredachi Informatsii*, pp. 9–16.

Kraskov, Alexander; Stögbauer, Harald; Grassberger, Peter (2004): Estimating mutual information. In *Phys. Rev. E* 69 (6 Pt 2), p. 66138. DOI: 10.1103/PhysRevE.69.066138.

Little, Max A.; McSharry, Patrick E.; Roberts, Stephen J.; Costello, Declan A. E.; Moroz, Irene M. (2007): Exploiting nonlinear recurrence and fractal scaling properties for voice disorder detection. In *Biomedical engineering online* 6, p. 23. DOI: 10.1186/1475-925X-6-23.

Lizier, Joseph T.; Prokopenko, Mikhail; Zomaya, Albert Y. (2012): Local measures of information storage in complex distributed computation. In *Information Sciences* 208, pp. 39–54. DOI: 10.1016/j.ins.2012.04.016.

Lorenz, Edward N. (1963): Deterministic nonperiodic flow. In *Journal of the atmospheric sciences*, 130-141.

Maris, Eric; Oostenveld, Robert (2007): Nonparametric statistical testing of EEG- and MEG-data. In *Journal of Neuroscience Methods* 164 (1), pp. 177–190. DOI: 10.1016/j.jneumeth.2007.03.024.

Marwan, Norbert; Kurths, Jürgen (2002): Nonlinear analysis of bivariate data with cross recurrence plots. In *Physics Letters A* 302 (5-6), pp. 299–307. DOI: 10.1016/S0375-9601(02)01170-2.

Marwan, Norbert; Wessel, Niels; Meyerfeldt, Udo; Schirdewan, Alexander; Kurths, Jürgen (2002): Recurrence-plot-based measures of complexity and their application to heart-rate-variability data. In *Phys. Rev. E* 66 (2 Pt 2), p. 26702. DOI: 10.1103/PhysRevE.66.026702.

Oostenveld, Robert; Fries, Pascal; Maris, Eric; Schoffelen, Jan-Mathijs (2011): FieldTrip: Open source software for advanced analysis of MEG, EEG, and invasive electrophysiological data. In *Computational Intelligence and Neuroscience* 2011, p. 156869. DOI: 10.1155/2011/156869.

Peng, C.-K.; Buldyrev, S. V.; Havlin, S.; Simons, M.; Stanley, H. E.; Goldberger, A. L. (1994): Mosaic organization of DNA nucleotides. In *Phys. Rev. E* 49 (2), pp. 1685–1689. DOI: 10.1103/physreve.49.1685.

Provenzale, A.; Smith, L. A.; Vio, R.; Murante, G. (1992): Distinguishing between low-dimensional dynamics and randomness in measured time series. In *Physica D: Nonlinear Phenomena* 58 (1-4), pp. 31–49. DOI: 10.1016/0167-2789(92)90100-2.

Ragwitz, M.; Kantz, H. (2002): Markov models from data by simple nonlinear time series predictors in delay embedding spaces. In *PHYSICAL REVIEW E* 6505 (5), p. 6201.

Romano, M. C.; Thiel, M.; Kurths, J.; Kiss, I. Z.; Hudson, J. L. (2005): Detection of synchronization for non-phase-coherent and non-stationary data. In *Europhys. Lett.* 71 (3), pp. 466–472. DOI: 10.1209/epl/i2005-10095-1.

Romano, M. Carmen; Thiel, Marco; Kurths, Jürgen; Bloh, Werner von (2004): Multivariate recurrence plots. In *Physics Letters A* 330 (3-4), pp. 214–223. DOI: 10.1016/j.physleta.2004.07.066.

Rosenstein, Michael T.; Collins, James J.; De Luca, Carlo J. (1993): A practical method for calculating largest Lyapunov exponents from small data sets. In *Physica D: Nonlinear Phenomena* 65 (1), pp. 117–134. DOI: 10.1016/0167-2789(93)90009-P.

Schreiber; Schmitz (1996): Improved Surrogate Data for Nonlinearity Tests. In *Physical review letters* 77 (4), pp. 635–638. DOI: 10.1103/PhysRevLett.77.635.

Schreiber, Thomas; Schmitz, Andreas (1997): Discrimination power of measures for nonlinearity in a time series. In *Phys. Rev. E* 55 (5), pp. 5443–5447. DOI: 10.1103/PhysRevE.55.5443.

Shannon CE, Weaver W. (1949): The mathematical theory of communication. In *Urbana*.

So; Ott; Schiff; Kaplan; Sauer; Grebogi (1996): Detecting unstable periodic orbits in chaotic experimental data. In *Physical review letters* 76 (25), pp. 4705–4708. DOI: 10.1103/PhysRevLett.76.4705.

Takens, Floris. (1981): Dynamical systems and turbulence. Detecting strange attractors in turbulence. Berlin, Heidelberg: Springer.

Takens, Floris. (1985): Dynamical systems and bifurcations. On the numerical determination of the dimension of an attractor. Berlin, Heidelberg: Springer.
